# Clinical Impact of Germline Multigene Sequencing in Pediatric Cohorts with a Wide Spectrum of Neoplasms

**DOI:** 10.3390/ijms27146395

**Published:** 2026-07-18

**Authors:** Vera Semenova, Elena Zhukovskaya, Ekaterina Zelenova, Valentina Kozlova, George Krasnov, Andrey Levashov, Garik Sagoyan, Tatiana Belysheva, Dmitriy Kharchikov, Amina Suleymanova, Natalia Ivanova, Anastasia Lozovaya, Marina Rubanskaya, Svetlana Gelfer, Elena Sharapova, Svetlana Mikhaylova, Timur Valiev, Alexander Karelin, Svetlana Varfolomeeva, Tatiana Nasedkina

**Affiliations:** 1 Engelhardt Institute of Molecular Biology, Russian Academy of Sciences, 119991 Moscow, Russia; sulpiridum@yandex.ru (V.S.); zelenovayeye@gmail.com (E.Z.); gskrasnov@mail.ru (G.K.); 2 N.N. Blokhin National Medical Research Centre of Oncology, Ministry of Health of the Russian Federation, 115478 Moscow, Russia; valentina-mk2011@yandex.ru (V.K.); andreyslevashov@mail.ru (A.L.); sagoyan-garik@mail.ru (G.S.); klinderma@bk.ru (T.B.); aminasuleymanova313@gmail.com (A.S.); nv.ivanova6@gmail.com (N.I.); ansti.loz@gmail.com (A.L.); marishvecova@yandex.ru (M.R.); sharapovae.v@yandex.ru (E.S.); astra-sn@mail.ru (S.M.); timurvaliev@mail.ru (T.V.); s.varfolomeeva@ronc.ru (S.V.); 3 Dmitry Rogachev National Medical Research Center of Pediatric Hematology, Oncology and Immunology, 117198 Moscow, Russia; elena_zhukovskay@mail.ru (E.Z.); dmitriy@dockharchikov.ru (D.K.); svetik1313@gmail.com (S.G.); alexandr.karelin@gmail.com (A.K.); 4 Department of Oncology, Sechenov First Moscow State Medical University, 119048 Moscow, Russia

**Keywords:** cancer predisposition syndrome, genetic testing, neoplasms, mutations, causative gene, adult-onset cancer syndrome

## Abstract

Cancer predisposition syndromes (CPSs) account for 8.5–18% of all childhood cancer cases. Most of them are inherited in an autosomal dominant pattern; consequently, there is a 50% risk of transmission to offspring. Early detection of CPSs is crucial for choosing patient treatment strategies and for counseling in the family. This study enrolled 886 pediatric patients with hematologic and solid neoplasms from prospective and retrospective cohorts (2018–2025). Clinical exome or multigene panel sequencing was used to analyze blood DNA. Overall, 186 pathogenic/likely pathogenic (PLP) variants in cancer-associated genes were identified in 176/886 (20%) of patients, and the most frequently mutated were the *NF1* (*n* = 35) and *TP53* (*n* = 18) genes. Among the 186 PLP variants, 126/886 (14.2%) were causative for pediatric neoplasms, while 56/886 (6.3%) were heterozygous mutations associated with adult-onset CPSs affecting DNA repair. The highest total mutation rate was revealed in retinoblastoma (80%), peripheral nerve sheath tumors (60%), and pheochromocytoma/paraganglioma (47%), while the lowest rate was found in hematologic malignancies (4.6%) and neuroblastoma (12%). The wide range and high frequency of deleterious variants in pediatric patients, especially in those with solid tumors, highlights the importance of multigene panel sequencing for the accurate determination of CPSs.

## 1. Introduction

Germline mutations in cancer predisposition genes are associated with a significant number of childhood cancer cases. According to the literature, the detection rate of pathogenic variants ranges from 8.5% to 18% [1,2,3,4,5,6,7,8] and depends on the spectrum of tumor types investigated, the diagnostic method (e.g., targeted gene panels or comprehensive whole-exome or whole-genome sequencing), patient selection based on criteria suggestive of a tumor syndrome, and the bioinformatic filtering criteria applied to genetic variants prior to statistical analysis. Timely diagnosis of cancer predisposition syndromes (CPSs) is an important task, as the identification of pathogenic variants can lead to changes in treatment protocols and initiate dynamic surveillance for the early detection of secondary tumors. In addition, this is of great importance for testing of close relatives, enabling the identification of a group at high risk of cancer and the development of measures for cancer prevention and early diagnosis in family members [2].

With the widespread introduction of NGS-based genetic testing into practice, it has become possible to simultaneously study a large number of genes in order to identify pathogenic variants associated with an increased risk of malignancies [9]. However, it remains unclear whether screening all children with neoplasms is cost-effective or whether genetic testing should be offered only to a specific cohort of patients. Currently, the 2016 Jongmans criteria are widely used to clinically assess the probability that a pediatric patient has a CPS. They include (1) a characteristic family history; (2) rare or specific tumor types; (3) the presence of primary multiple tumors, including bilateral or multifocal tumors; (4) phenotypic features (congenital malformations, skin manifestations, etc.); and (5) excessive treatment-related toxicity [10]. Fulfilling only one of these criteria is deemed sufficient for offering genetic analysis in pediatric cancer cases. Goudie et al. [11] propose a similar McGill Interactive Pediatric Onco-Genetic Guidelines (MIPOGG) algorithm for referring patients for genetic testing.

However, these criteria are not universal for all CPSs. For example, DICER1 syndrome is characterized by incomplete penetrance (the risk of developing tumors by age 50 is 19.3%), and in 13% of cases, mutations arise de novo. Accordingly, the patient may not have affected relatives. Furthermore, this syndrome lacks specific phenotypic manifestations and developmental anomalies, and the first clinical manifestation in some cases is a neoplasm that is not classified as a “high-risk” tumor (multinodular goiter, thyroid adenoma, etc.) [12]. Specialized diagnostic criteria exist for many syndromes associated with pediatric malignancies, including neurofibromatosis type 1, Gorlin-Goltz syndrome, Cowden syndrome, and DICER1 syndrome, among others [13,14,15,16]. At the same time, approximately 100 tumor syndromes with manifestation in childhood have been described to date, which significantly complicates clinical diagnosis [7].

Another controversial issue is the required scope of genetic testing. On the one hand, genome or exome sequencing allows for the investigation of the largest possible number of genes associated with cancer development. Compared to small panels, exome sequencing demonstrates higher diagnostic efficacy [6]. Whole-genome sequencing enables the identification of pathogenic variants in intronic regions and the detection of chromosomal rearrangements using bioinformatic methods without additional diagnostic approaches. Thus, 5–11% of neurofibromatosis type 1 cases are associated with large deletions in the *NF1* gene, which cannot be detected by examining only the coding regions of the gene [17]. In addition, large-scale paired tumor and germline sequencing is rapidly being adopted in pediatric oncology [18].

On the other hand, extensive genetic testing increases the likelihood of detecting variants of uncertain significance and incidental findings in genes not associated with cancer, which complicates the clinical interpretation of the results [2,6]. For this reason, multigene panel sequencing has become widespread, allowing for the study of target regions with high coverage. Sequencing depth can be important for detecting low-grade mosaicism, which occurs in syndromes such as neurofibromatosis type 1, hereditary retinoblastoma, tuberous sclerosis, and others [19]. Currently, there is no single standard for the composition of panels used to examine pediatric cancer patients [8]. Thus, most panels for diagnosing cancer predisposition include genes for hereditary syndromes that manifest in adulthood (*BRCA1*, *BRCA2*, *PALB2*, *ATM*, *CHEK2*, *MSH2*, *MSH6*, *MLH1*, and *PMS2*). Current recommendations question the relevance of testing these genes in childhood [20,21,22]; however, through further screening of family members, there is a possibility of identifying similar variants in older relatives, who will then require immediate dynamic monitoring. In addition, homozygous and compound heterozygous mutations in the *MSH2*, *MLH1*, *PMS2*, and *MSH6* genes are associated with constitutional mismatch repair deficiency (CMMRD) syndrome, which carries a high risk of childhood malignancies [23].

A number of studies have compared approaches to genetic testing: either a phenotype-first approach, where genetic testing is based on clinical suspicion of CPS, or a genotype-first approach, which involves non-selective testing of all pediatric patients with neoplasms [2,9,24]. Overall, genetic testing has been shown to be highly effective in children meeting specific clinical criteria, particularly in definite tumor groups such as retinoblastoma and central nervous system (CNS) tumors [9,24]. However, further studies involving large, ethnically diverse cohorts are important to evaluate the causality between germline findings and tumor development and to expand the scientific basis for personalized treatment strategies. 

The primary aim of our study was to compare the results of germline sequencing of childhood cancer predisposition genes with those of genetic testing based on clinical selection criteria in prospective and retrospective cohorts of Russian children and adolescents with neoplasms of various locations. The results of genetic testing using a multigene panel or clinical exome sequencing were compared with clinical criteria for CPS eligibility. The obtained data allow us to evaluate the need for screening all children with cancer and to discuss the appropriate scope of genetic testing for effective CPS diagnosis.

## 2. Results

### 2.1. Patient Cohorts

**Figure 1:**
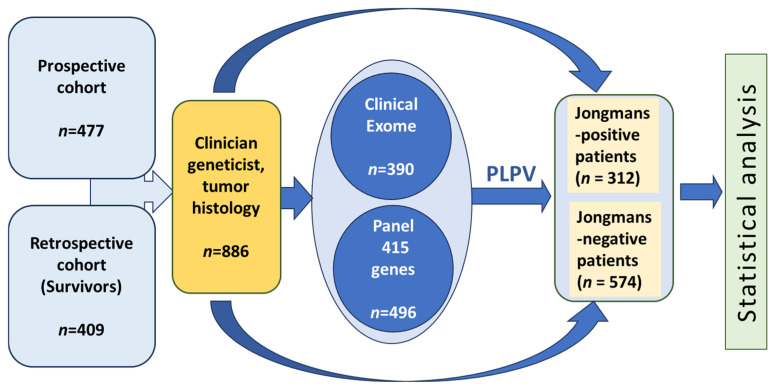
The design of the study. PLPV—pathogenic/likely pathogenic variants.

This study assembled genetic and phenotypic data from two cohorts: a prospective cohort of children with various neoplasms who were referred to the N.N. Blokhin National Medical Research Centre of Oncology, and a retrospective cohort of childhood cancer survivors from the Rehabilitation Department of the Dmitry Rogachev National Medical Research Center. Patient recruitment and testing were conducted from September 2018 to May 2025. All patients were evaluated by a clinical geneticist, and comprehensive sequencing of peripheral blood DNA was performed using either the TruSight One Expanded panel (Illumina), which includes 6794 genes, or a custom panel of 415 cancer-associated genes. The study design is presented in Figure 1.

In total, 886 children with hematologic malignancies and solid neoplasms of various localizations, aged 0 to 18 years at diagnosis, were enrolled; among them were 495 girls (56%) and 391 boys (44%). The median age at disease manifestation was 6 years (range, 0 to 17 years). Among them, 855 patients had only one primary tumor (either bilateral tumors of the same type, or multiple foci of the same tumor, or relapse), 28 patients had two different primary tumors, and 3 patients developed three primary malignancies of different histology and localization. Clinical characteristics of the patients from both cohorts are presented in Table 1 and Table S1.

**Figure 2:**
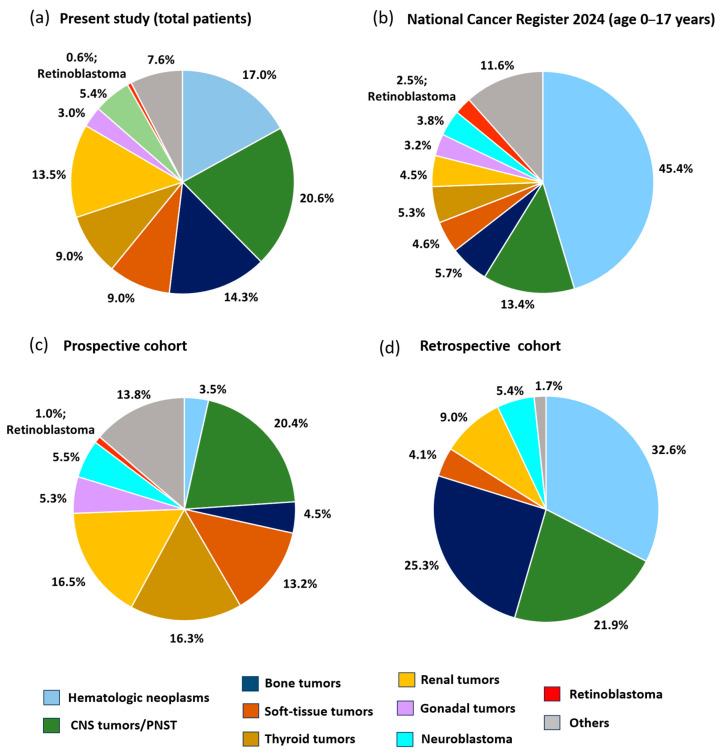
Pediatric cancer diagnosis distributions. (**a**)—The present study (*n* = 886), (**b**)—the cohort of patients aged from 0 to 17 years registered with cancer in Russia in 2024 (*n* = 3792), (**c**)—the prospective cohort (*n* = 477), (**d**)—the retrospective cohort (*n* = 409). Percentage of neoplasms in each cohort is displayed in the respective pie chart.

Compared with the distribution of childhood cancers in the general Russian population (according to the 2024 report of the Russian Cancer Registry [25]), our sample had a significantly lower proportion of hematologic malignancies (17% vs. 45.4%) and a significantly higher proportion of solid neoplasms, including CNS/peripheral nerve sheath tumors (PNST) (20.6% vs. 13.4%), bone tumors (14.3% vs. 5.7%), soft tissue tumors (9% vs. 5.3%), and renal tumors (13.5% vs. 4.5%) (Figure 2a,b). Furthermore, there was a noticeable difference in the proportion of patients with various diagnoses between the two cohorts: in the retrospective cohort, compared with the prospective one, hematologic neoplasms and bone tumors were overrepresented (32.6% vs. 3.5%, and 25.3% vs. 4.5%, respectively) (Figure 2c,d).

### 2.2. Multigene Panel Sequencing

The clinical exome was analyzed in 390/886 (44%) patients, and targeted sequencing of the coding regions of 415 cancer-associated genes was performed in 496/886 (56%) patients. Sequencing data were thoroughly investigated, and pathogenic/likely pathogenic (PLP) variants in genes associated with the development of various neoplasms were identified. Genetic data were compared with clinical diagnosis and tumor histology. Family history was also analyzed, and segregation analysis was performed in cases where samples from close relatives were available. Fulfillment of the Jongmans criteria [10] was assessed retrospectively by review of medical records including the clinical geneticist’s report. Criteria 1–4 were addressed in our study, whereas criterion 5 was not applied due to the lack of objective data on treatment toxicity in these cohorts (Table S1). In addition, an expanded list of neoplasm types (criterion 2) was used, including those recommended by MIPOGG [11] (Table S2). 

### 2.3. Spectrum of Genetic Variation and Detected Variants

**Figure 3:**
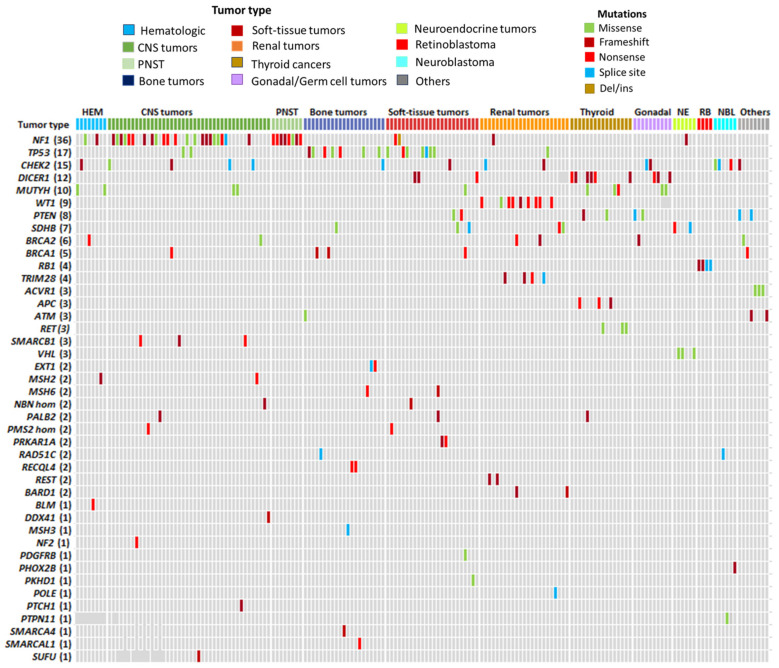
Mutational landscape of pediatric population with different neoplasms. HEM—hematologic neoplasms, CNS—central nervous system, PNST—peripheral nerve sheath tumors, NBL—neuroblastoma, NE—neuroendocrine tumors, RB—retinoblastoma.

Pathogenic and likely pathogenic variants (*n* = 186) were detected in 176/886 (20%) patients in 42 different cancer-associated genes: *ACVR1*, *APC*, *ATM*, *BARD1*, *BLM*, *BRCA1*, *BRCA2*, *CHEK2*, *DDX41*, *DICER1*, *EXT1*, *MSH2*, *MSH3*, *MSH6*, *MUTYH*, *NBN*, *NF1, NF2*, *PALB2*, *PDGFRB*, *PHOX2B*, *PKHD1*, *PMS2*, *POLE, PRKAR1A*, *PTCH1*, *PTEN*, *PTPN11*, *RAD51C*, *RB1*, *RECQL4*, *REST*, *RET*, *SDHB*, *SMARCA4*, *SMARCAL1*, *SMARCB1*, *SUFU*, *TP53*, *TRIM28*, *VHL*, *WT1*. The highest number of mutations were identified in the genes *NF1* (*n* = 36), *TP53* (*n* = 17), *CHEK2* (*n* = 15) and *DICER1* (*n* = 12) (Figure 3, Table S3).

Two PLP variants in different genes were identified simultaneously in 6/176 (3.4%) patients, and three PLP variants were found in 2/176 (1%) patients (Figure 3, Tables S3 and S4). Among the 186 identified variants, 32% (*n* = 59) were frameshift mutations, 27.0% (*n* = 51) were nonsense mutations, 29% (*n* = 54) were missense mutations, 11.5% (*n* = 21) were splice site variants, and 0.5% (*n* = 1) was in-frame deletion. In total, 167 (90%) PLP variants were associated with autosomal dominant syndromes; 15 (8%) heterozygous PLP variants were assigned to autosomal recessive inheritance and could be considered carrier status, and in 4 (2%) cases, homozygous mutations related to autosomal recessive syndromes were identified: CMMRD (*PMS2* mutations, two patients), and Nijmegen syndrome (*NBN* mutation, two patients). In total, 33/186 (18%) of the identified variants had not been previously described in ClinVar but were pathogenic/likely pathogenic according to American College of Medical Genetics and Genomics (ACMG) criteria.

Segregation analysis was performed for 49/176 (28%) patients with identified PLP variants. In 76% (38/49) of cases, clinically significant variants were inherited from one of the parents, and in 24% (11/49) of cases, de novo mutation status was confirmed (Table S3).

### 2.4. Tumor Spectrum in Carriers of Pathogenic Mutations in Cancer-Associated Genes

**Figure 4:**
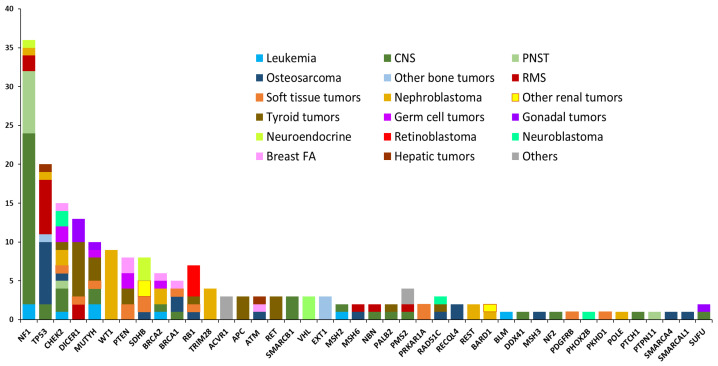
Tumor spectrum associated with different affected genes. CNS—central nervous system, PNST—peripheral nerve sheath tumors, RMS—rhabdomyosarcoma, FA—fibroadenoma.

PLP variants within the same gene were associated with different tumor types. We observed the broadest tumor spectrum for the *CHEK2* gene (10 different tumor types), *TP53*, *MUTYH*, and *NF1* (6 tumor types each), as well as *BRCA2* (5 types), and *BRCA1*, *SDHB*, *RB1, PTEN*, and *DICER1* (4 types each). At the same time, mutations in the *WT1*, *TRIM28*, and *REST* genes were noted only in nephroblastoma, *RET* and *APC* mutations in thyroid cancer, and *VHL* mutations were observed exclusively in pheochromocytoma (Figure 4).

**Figure 5:**
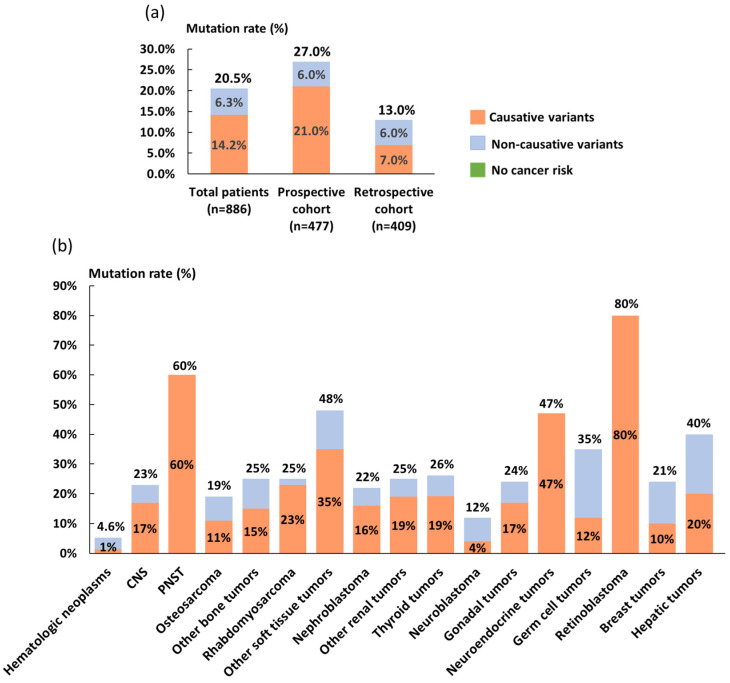
Distribution of PLP variants of different clinical significance: (**a**)—within different cohorts (total number of patients, *n* = 886); and (**b**)—within each type of tumor (total number of tumors, *n* = 920) (CNS—central nervous system, PNST—peripheral nerve sheath tumor).

The overall mutation rate varied significantly among patient groups with different tumor types. To more accurately assess the clinical significance of the identified variants and to conduct a detailed analysis of the associations between gene mutations and tumor type, we categorized the PLP variants using the criteria described previously: (1) “high-risk” or “low to moderate risk” of cancer, and (2) “matching” or “non-matching” of the tumor phenotype with previously reported phenotypes associated with pathogenic variants in this gene [7]. Combining these definitions, we considered causative variants to be associated with a high risk of cancer development or uncontrolled tumor growth and with a phenotype (tumor type) previously described for this gene in at least two patients [7]. Non-causative variants or “secondary findings” were defined as heterozygous variants in genes of dominant adult-onset cancer syndromes either of “high risk” (*BRCA1*, *BRCA2*, *PALB2*, *BARD1*, *MSH2*, *MSH6*, *PMS2, POLE*, and *DDX41*), or “low/moderate risk” (*CHEK2*, *ATM*, and *RAD51C*), as well as heterozygous variants in genes for autosomal recessive cancer syndromes (*BLM*, *MUTYH*, *MSH3*, and *RECQL4*), which were associated with a “non-matching” childhood cancer phenotype. PLP variants in genes *ACVR1* and *PKHD1* were considered not to be associated with hereditary cancer (Table S5, Figure 5).

Germline PLP variants were identified in 20.5% (186/886) of pediatric patients. The share of PLP variants in genes causative for pediatric CPSs, which correspond to known and well-described phenotypes, accounted for 14.2% (126/886). Heterozygous variants with non-matching phenotypes, mainly represented by genes of the DNA repair system, associated with adult-onset autosomal dominant and autosomal recessive cancer syndromes, amounted to 6.3% (56/886). Another 0.5% (4/886) harbored PLP variants associated with other hereditary diseases with clinical manifestations similar to cancer (fibrous dysplasia and polycystic kidney disease) (Figure 5a). 

When comparing the distribution of PLP variants between different tumor types, the lowest frequency was detected in hematologic neoplasms and neuroblastoma (overall frequency of 4.6% and 12%, and for matching phenotypes of 1% and 4%, respectively). The highest frequency of mutations in pediatric CPS causative genes was observed in retinoblastoma (80%), tumors of the peripheral nervous system (60%) and neuroendocrine tumors (pheochromocytoma and paraganglioma) (47%) (Figure 5b). We found that the total number of PLP variants was significantly higher in the prospective cohort, compared with the retrospective one (130/477, 27% vs. 52/409, 13%, OR = 2.6; 95% CI: 1.8–3.7, *p* < 0.0001) (Table S6). Since the cohorts differed notably in the representation of individual tumor types with various frequencies of PLP variants, we also compared separate tumor groups that were more equal in terms of patient numbers. The rate of PLP variants was higher in the prospective cohort in CNS tumors (22/89, 25% vs. 19/90, 21%), rhabdomyosarcomas (12/44, 27% vs. 4/16, 25%), and nephroblastomas (19/84, 23% vs. 4/36, 11%), but the difference was not significant (Table S6). No significant difference was found in the spectrum and frequency of PLP variants between the two approaches: clinical exome sequencing and targeted 415-gene panel sequencing (Tables S7 and S8).

### 2.5. Spectrum of Germline PLP Variants in Patients with Different Neoplasms 

In hematologic malignancies, clinically significant variants were identified in the *NF1* gene (*n* = 2). In CNS tumors, the majority of causative variants were also found in the *NF1* gene (20/179, 11%), including patients diagnosed with optic pathway glioma (OPG) (*n* = 10), astrocytoma (*n* = 5), other gliomas (*n* = 2), ependymoma (*n* = 1), intracranial neurofibroma (*n* = 1), and atypical teratoid/rhabdoid tumor (AT/RT) of the frontotemporal lobe (*n* = 1). Other cases of AT/RT (3/179, 2%) were associated with PLP variants in the *SMARCB1* gene. Mutations in the *TP53* gene were detected in two patients with choroid plexus carcinoma, and mutations in the *PTCH1* and *SUFU* genes associated with Gorlin–Goltz syndrome were detected in two cases of medulloblastoma. A homozygous mutation in *PMS2* (CMMRD syndrome) was identified in a patient with an astrocytoma, and a homozygous mutation in the *NBN* gene, c.657_661del (p.Lys219fs), in a patient with an embryonal tumor of cerebellum. A mutation in the *NF2* gene was associated with multiple neurofibromas and a meningioma. Additionally, PLP variants in the *NF1* gene were found in 9/15 (60%) of patients with peripheral nervous system tumors: neurofibromas, malignant peripheral nerve sheath tumors (MPNSTs), schwannomas, and plexiform neurofibroma (Table S5). 

In osteosarcomas, the most frequently affected gene was *TP53* (8/107, 7.5%). Variants in other genes associated with dominant tumor syndromes, *SDHB* (*n* = 1) and *RB1* (*n* = 1), were also found. A patient with conventional chondroblastic osteosarcoma had a variant c.2542G>T (p.Glu848*) in the *SMARCAL1* gene. The overall mutation rate was 19/107 (19%), but causative variants accounted for 11% of cases. In other bone tumors, PLP variants were found in 5 of 20 cases (25%). Two patients with hereditary multiple exostoses (osteochondromas) (HME) had a mutation in the *EXT1* gene. One of these patients developed Ewing sarcoma as a second tumor [26]. It should be noted that in the remaining 13 cases of Ewing sarcoma, germline mutations were not detected. 

In rhabdomyosarcomas (RMSs), germline PLP variants were detected in 15/61 (25%) of cases. Rhabdomyosarcomas of various locations were predominantly represented by the histological subtype embryonal rhabdomyosarcoma (eRMS), and one case was a pleomorphic rhabdomyosarcoma. Mutations in the *TP53* gene were the most frequent events (6/61, 10%). In three patients, *NF1* variants were detected. Two of them had multiple café-au-lait spots, while a patient with eRMS of parameningeal localization and renal carcinoma as a second tumor had an attenuated phenotype. Other findings were a variant in the *DICER1* gene, a homozygous mutation in the *PMS2* gene (CMMRD), and a homozygous *NBN* mutation c.657_661del (p.Lys219fs) (Nijmegen syndrome).

Other soft tissue tumors included both benign and malignant neoplasms. Two patients with lipoma and a specific phenotype (macrosomia, macrocephaly, and prominent frontal bossing) had mutations in the *PTEN* gene (Cowden syndrome). Two other patients with myxoid tumors and multiple intradermal nevi had heterozygous mutations in the *PRKAR1A* gene (Carney complex). Three mutations in the *PDGFRA*, *BRCA1*, and *MUTYH* genes were detected in a patient with infantile myofibromatosis. Analysis of a tumor tissue sample revealed a second pathogenic mutation in the *PDGFRB* gene suggesting a role for this gene in the disease development. Mutations in the *SDHB* gene (*n* = 2) were associated with gastrointestinal stromal tumor (GIST). A *DICER1* variant was found in a patient with high-grade endometrial stromal sarcoma (ESS). Overall mutation rate was 11/23 (48%), and causative variants accounted for 35%.

In patients with nephroblastoma, the most frequently detected mutations were in the *WT1* (9/105, 9%), *TRIM28* (4/105, 4%), and *REST* (2/105, 2%) genes [27], and rare isolated findings were also found—mutations in *TP53* and *NF1*. Other renal tumors consisted mainly of renal cell carcinoma (RCC) (13/17, 76%), rhabdoid tumors (2/17, 12%), and single cases of clear cell sarcoma and angiomyolipoma. Two *SDHB* mutations were detected in patients with succinate dehydrogenase-deficient renal cell carcinoma. A patient with papillary renal cell carcinoma had the same mutation in the *BARD1* gene c.2300_2301del (p.Val767AspfsTer4), as the patient with nephroblastoma. Genomic profiling of tumor tissue did not reveal other significant variants, the *BARD1* mutation was present in a heterozygous state with a VAF of 53.8%, and the tumor mutation burden (TMB) was 2 mut/Mb.

Among patients with thyroid neoplasms, medullary cancer was detected in three cases (3/85, 3.5%), and all three patients had hot-spot mutations in the *RET* gene: c.2753T>C (p.Met918Thr) in exon 16, and c.1900T>C (p.Cys634Arg) in exon 11. Mutations in the *DICER1* gene were most frequent (7/85, 8.2%), and associated with papillary or follicular carcinoma, as well as with follicular adenoma. Mutations in the *PTEN* gene were detected in two patients (2/85, 2.3%) with phenotypic features of Cowden syndrome, and one of them had intraductal papillomas in both breasts. A patient with retinoblastoma and an *RB1* mutation developed papillary thyroid carcinoma as a second tumor. Mutations in the *APC* gene were detected in three cases (3/85, 3.5%), and in one of them, a rare tumor subtype, cribriform-morular thyroid carcinoma, was identified. 

Of the 50 neuroblastoma/ganglioneuroma cases, one patient had a PLP variant in the *PHOX2B* gene. Another patient with ganglioneuroma, short stature, development delay, amblyopia, astigmatism, and facial dysmorphism, was found to have the c.1472C>A (p.Pro491His) mutation in the *PTPN11* gene. Gonadal tumors were identified in 29 patients and included a wide range of ovarian neoplasms (*n* = 23), as well as three testicular tumors. Among the 14 patients with sex cord stromal tumors (SCST), including Sertoli-Leydig cell and juvenile granulosa cell tumors, three carried mutations in the *DICER1* gene (3/14, 21%). Additional pathogenic variants included a *PTEN* mutation in a patient with embryonal ovarian carcinoma, macrosomia, scaphocephaly, and lipoma; and a *SUFU* mutation in a patient with ovarian fibromas. 

Neuroendocrine tumors were represented by pheochromocytomas (*n* = 10), paragangliomas (*n* = 4), and gastric neuroendocrine tumor (*n* = 1). Among patients with pheochromocytoma/paraganglioma (*n* = 14), *VHL* and *SDHB* mutations were each found in three cases. Pheochromocytoma was also detected in a patient with *NF1* mutation and neurofibromatosis type 1. The overall mutation rate of germline CPS variants was 7/15 (47%). In extragonadal germ cell tumors, only one mutation in the *PTEN* gene (1/8, 12%) was detected. Our sample included five patients with retinoblastoma, four of whom (80%) had mutations in the *RB1* gene. In one case, a patient with an *RB1* mutation developed a second primary tumor, osteosarcoma, and in another patient, two second tumors (papillary thyroid carcinoma and rhabdomyosarcoma) were detected.

Breast tumors were fibroadenomas (*n* = 27), phyllodes tumors (*n* = 2) and intraductal papilloma (*n* = 1). The age at onset of breast tumors ranged from 13 to 17 years. Mutations in the *PTEN* gene (*n* = 3) were associated with multiple fibroadenomas or multiple intraductal papillomas. In patients with isolated fibroadenomas, mutations in *BRCA1* (*n* = 1) and *BRCA2* (*n* = 1) were found [28]. Among patients with hepatic tumors (*n* = 5), one *TP53* mutation was identified in a patient with hepatocellular carcinoma as a second tumor in Li-Fraumeni syndrome. No mutations were detected in two cases of rhabdoid tumor of the liver.

In total, germline causative variants were found in 24 genes associated with well-known pediatric CPSs. Other variants in 16 genes (*ATM*, *BARD1*, *BLM*, *BRCA1*, *BRCA2*, *CHEK2*, *DDX41*, *MSH2*, *MSH6*, *MUTYH,* etc.) were considered non-causative events that had no direct association with the patient phenotype. The frequencies of non-causative variants varied significantly in different types of neoplasms: from 0% in retinoblastoma, PNST, and neuroendocrine tumors, to 13% in soft-tissue tumors, and 23% in extra-gonadal germ cell tumors. 

Three patients with pathologic soft tissue growth were found to have the same heterozygous variant c.617G>A (p.Arg206His) in the *ACVR1* gene and were diagnosed with fibrodysplasia ossificans progressiva. Before genetic testing, one patient at 6 months of age had undergone surgical excision of a fibrous proliferative mass that was classified as chondromyxoid fibroma, while another patient was diagnosed with infantile myofibromatosis at the age of one year.

### 2.6. Primary Multiple Tumors

**Figure 6:**
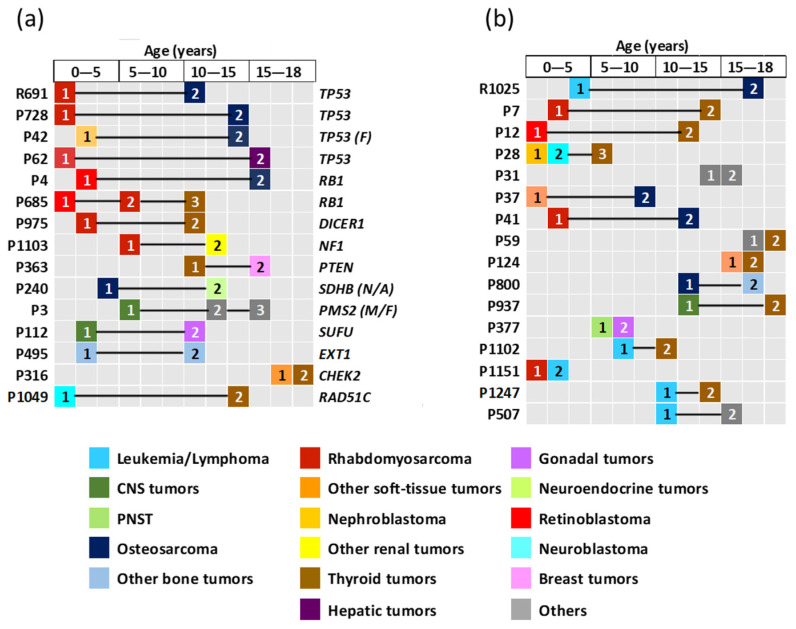
Tumor type and age of onset in patients with primary multiple neoplasms: (**a**)—patients with mutations in cancer-associated genes; (**b**)—patients without mutations.

Thirty-one patients (31/886, 3.5%) had more than one primary malignancy, with 28 patients (28/886, 3.2%) having two tumors and 3 patients (3/886, 0.3%) having three primary tumors, for a total of 65 tumors (Figure 6). In this group, mutations in cancer-associated genes were detected in 15 patients (15/31, 48%). Variants associated with pediatric CPSs (matching phenotypes) were 13/31 (42%), which is significantly more common than in patients with only one tumor (13%, 113/886; OR, 4.84; 95% CI, 2.31–10.15; *p* < 0.0001). The highest frequency of identified PLP variants was noted in the *TP53* (4/15, 27%) and *RB1* (2/15, 13.5%) genes. In the group of patients without mutations, there were more cases of leukemia/lymphoma compared to patients with mutations (5/16, 31% vs. 0/15, 0%, OR, 14.8; 95% CI, 0.74–296; *p* = 0.04), as well as cases of thyroid cancer (8/16, 50% vs. 5/15, 33%; *p* > 0.05).

It should be noted that only 2/3 (66%) of patients with more than two primary tumors were found to have a causative variant: a homozygous mutation in the *PMS2* gene in patient P3 and a heterozygous pathogenic variant in the *RB1* gene in patient P685. In one case (patient P28) who had synchronously developed neuroblastoma and nephroblastoma followed by papillary thyroid cancer, no mutation was detected. Rare combinations of primary tumors were also identified: nephroblastoma and osteosarcoma in patient P42 (mutation *TP53*), embryonal rhabdomyosarcoma and translocation renal cell carcinoma in patient P1103 (mutation *NF1*), and osteosarcoma and pheochromocytoma in patient P240 (mutation *SDHB*) (Figure 6).

### 2.7. The Fulfillment of Jongmans Criteria and Germline Mutation Rate

**Figure 7:**
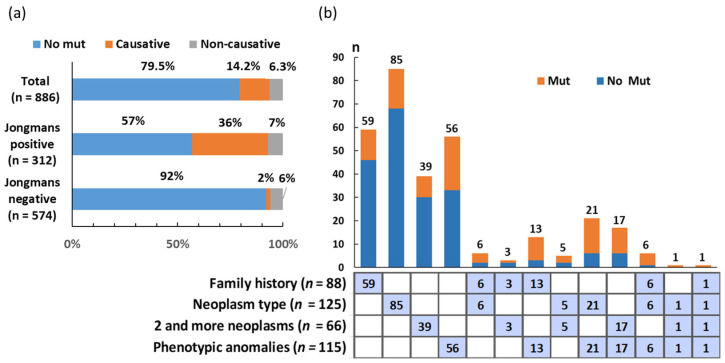
Jongmans criteria and the probability of detecting PLP variants in patients with various neoplasms. (**a**)—Frequency of causative and non-causative variants among patients meeting the Jongmans criteria (positive) and among patients not meeting any of the criteria (negative); (**b**)—Number of patients with various criteria and their combinations in the Jongmans positive group, with and without causative mutations. Here, criterion “2 and more neoplasm” indicates the presence of bilateral, multifocal, or metachronous primary neoplasms.

We performed a retrospective analysis of patient clinical characteristics according to the modified Jongmans criteria (Tables S1 and S2). The Jongmans-positive group included 312/886 (35%) patients who met at least one of the criteria, while the Jongmans-negative group consisted of 574/886 (65%) patients. We then assessed the proportion of causative and non-causative PLP variants in each of these groups. While the proportion of causative PLP variants was 14.2% in the overall group, it increased to 36% in the Jongmans-positive group and decreased to 3% in the Jongmans-negative group. The percentage of non-causative variants was approximately the same in both groups, 7% and 6%, respectively (Figure 7a). 

We also assessed the association between individual Jongmans criteria, as well as combinations of criteria, and the presence of mutations in CPS genes. The greatest contribution was made by phenotypic features, which were noted in a total in 115 patients, and mutations in CPS genes were detected in 47% of these patients (56/115) compared to 14.2% in the overall group (126/886) (OR = 5.725, 95% CI: 3.79–8.64, *p* < 0.0001, Fisher’s exact test). In the total sample of 886 patients, family history of cancer data was available for 96% (*n* = 851/886) of patients. Overall, 53% (*n* = 451/851) of patients had first- to fourth-degree relatives diagnosed with cancer. However, only 10.3% (*n* = 88/851) of patients met the Jongmans criterion for family history. Among patients with this criterion, causative PLP variants were identified in 39% (34/88) vs. 15% (125/851) (OR = 3.657, 95% CI: 2.29–5.85, *p* < 0.0001). The “neoplasm type” and “2 or more neoplasms” criteria also showed a statistically significant association with the presence of a mutation in the patients’ genotypes (46/125, 37% vs. 126/886, 14.2%; OR = 3.512, 95% CI: 2.33–5.29, *p* < 0.0001, and 26/66, 39% vs. 126/886, 14.2%; OR = 3.921, 95% CI: 2.31–6.65, *p* < 0.0001, respectively). 

Patients who met only one of the Jongmans criteria constituted the majority (76%, 239/312), those meeting two criteria were 21% (65/312), and those meeting three or more criteria together were 3% (8/312). Simultaneous fulfillment of two or more Jongmans criteria significantly increased the likelihood of the presence of a causative CPS mutation (Figure 7b).

However, thirteen cases with genetically confirmed CPS did not meet any of the Jongmans criteria examined, including six patients with Li-Fraumeni syndrome (*TP53* mutations), three patients with DICER1 syndrome (*DICER1* mutations), two patients with nephroblastoma (*WT1* mutations), a patient with neuroblastoma (*PHOX2B* mutation), and another patient with osteosarcoma (*SMARCAL1* mutation).

### 2.8. Statistical Analysis Using Multivariate Logistic Regression

**Figure 8:**
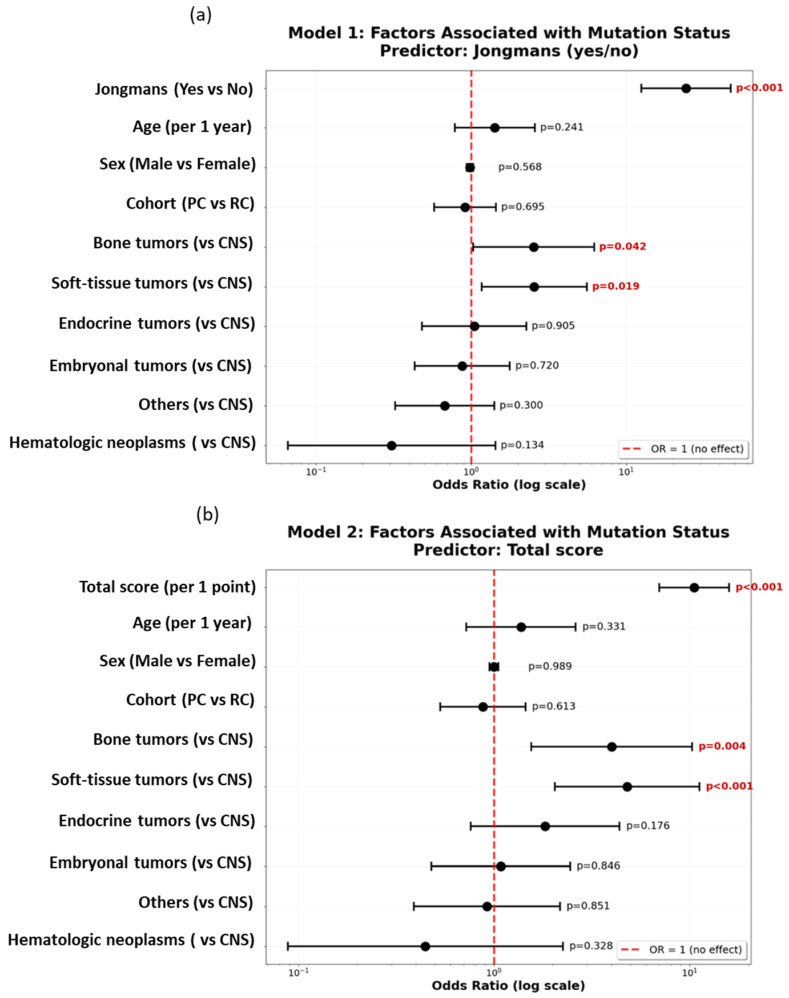
Multivariable logistic regression. (**a**)—binary Model 1 accounting to the presence or absence of Jongmans criteria (yes/no); (**b**)—continuous Model 2, taking into account total Jongmans score (from 0 to 4). Tumor type: CNS tumors (*n* = 179), hematologic neoplasms (*n* = 151), bone tumors (osteosarcomas, Ewing sarcoma, osteochondromas) (*n* = 119), soft-tissue tumors (rhabdomyosarcoma, other soft-tissue tumors) (*n* = 80), endocrine tumors (thyroid carcinoma, pheochromocytoma/paraganglioma, gastric neuroendocrine tumor) (*n* = 94), embryonal tumors (nephroblastoma, neuroblastoma, hepatoblastoma, retinoblastoma) (*n* = 155), other tumors (*n* = 108). Cohort type: prospective (PC) and retrospective (RC). If a patient had several neoplasms of different locations, only the first tumor was taken into account. Statistically significant *p*-values are marked in red.

Two multivariable logistic regression models were designed to evaluate the association between phenotypic features (Jongmans criteria) and the presence of germline mutations. The following clinical characteristics were included in the analysis: sex, age at diagnosis, tumor type, and cohort type (prospective, PC or retrospective, RC). All neoplasms were combined into seven groups: CNS tumors, bone tumors, soft-tissue tumors, embryonal tumors, hematologic neoplasms, endocrine tumors, and other tumors. Central nervous system (CNS) tumors were selected as the reference, as they represent the most numerous and clinically homogeneous group, ensuring statistical stability of the estimates and enabling interpretation of results compared with other tumor types. The first model (1) used a binary predictor (meeting or not meeting at least one Jongmans criterion), and the second (2) used a quantitative predictor (total score for the Jongmans criteria) (Figure 8a,b; Table S9).

In Model 1, the presence of Jongmans criteria was the strongest independent predictor of detecting a germline CPS mutation in the patient’s genotype (OR = 24.310; 95% CI: 12.544–47.112, *p* < 0.001). Model 2 revealed a dose-dependent effect: each additional point on the total score increased the odds of detecting a mutation 10-fold (OR = 10.694; 95% CI: 7.095–16.120; *p* < 0.001).

Tumor type was a significant independent predictor in both models. In Model 1, bone tumors (OR = 2.533; 95% CI: 1.034–6.205; *p* = 0.042) and soft tissue tumors (OR = 2.553; 95% CI: 1.169–5.576; *p* = 0.019) were associated with significantly higher odds of detecting mutations compared with CNS tumors. In Model 2, these associations persisted and became stronger: for bone tumors OR = 4.048; 95% CI: 1.570–10.43; *p* = 0.004, and for soft tissue tumors OR = 4.825; 95% CI: 2.059–11.308; *p* < 0.001, respectively. 

To evaluate the discriminative power of the models, an ROC-AUC analysis was performed, and a 10-fold cross-validation was conducted. The mean cross-validated AUC was 0.8491 (SD = 0.0427) for Model 1 and 0.8860 (SD = 0.0453) for Model 2, confirming the strong predictive performance of the latter. To check for multicollinearity between predictors, the Variance Inflation Factor (VIF) was calculated. The maximum VIF value in both models was 3.5, indicating the absence of significant multicollinearity (all VIF values were <5; Table S9). 

Both models demonstrated high sensitivity (89.9% and 88.8%, respectively), supporting their utility in selecting pediatric patients for genetic testing aimed at detecting CPS mutations. The models achieved specificities of 74.2% and 75.9%, respectively, which are acceptable for screening tests and indicate a moderate false-positive rate. The low positive predictive values (PPV: 36.5% and 37.9%) are attributable to the low mutation prevalence in the study cohort (14.2%), a characteristic feature of screening tools designed to detect rare events. In contrast, the high negative predictive values (NPV: 97.8% and 97.5%) represent a key finding, enabling confident exclusion of mutations in patients who screen negative. This suggests that the developed models can serve as a screening instrument to identify low-risk patients. For overall evaluation and comparison of the models, the Akaike information criterion (AIC) was calculated. A comparison of AIC values confirms a clear superiority of Model 2 over Model 1: AIC_1_ = 533.510 for Model 1 and AIC_2_ = 479.339 for Model 2 (ΔAIC = AIC_1_ − AIC_2_ = 54.171). A ΔAIC > 10 is strong evidence of substantially better goodness-of-fit and higher predictive value of the model with the quantitative predictor.

## 3. Discussion

### 3.1. Total Diagnostic Yield and Comparison with Other Studies

**Figure 9:**
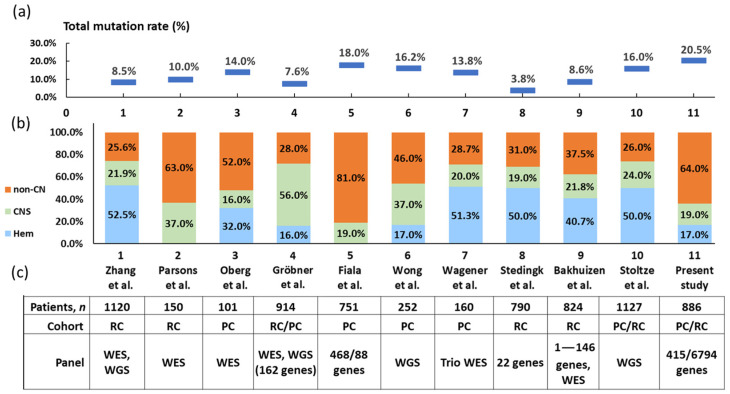
Frequency of PLP variants associated with hereditary tumor syndromes among pediatric patients across different studies. (**a**)—total mutation rate, (**b**)—sample composition, (**c**)—other characteristics (number of patients, type of cohort, method). Hem—hematologic malignancies, CNS—central nervous system, WES—whole exome sequencing, WGS—whole genome sequencing. 1—Zhang et al. [1], 2—Parsons et al. [6], 3—Oberg et al. [29], 4—Gröbner et al. [30], 5—Fiala et al. [3], 6—Wong et al. [5], 7—Wagener et al. [4], 8—Stedingk et al. [31], 9—Bakhuizen et al. [2], 10—Stoltze et al. [7], 11—Present study.

This study included 886 children with hematological malignancies and solid tumors. Clinically significant (likely pathogenic and pathogenic) genetic variants predisposing to malignancy were identified in 20.5% of patients, which is higher than previously reported values. For comparison, the frequencies of clinically significant variants associated with cancer predisposition syndromes identified in various studies of pediatric patients are presented [1,2,3,4,5,6,7,29,30,31] (Figure 9, Table S10).

In our study, the frequency of clinically significant variants may be higher than in other studies, in part due to biased selection of patients by oncologists for referral to genetic counseling and subsequent genetic testing, especially in the prospective cohort. The sample composition is also of great importance, namely, the proportion of patients with hematologic malignancies, in which the mutation rate is significantly lower than in non-hematologic solid neoplasms. Our study is not population-based, and the distribution of patients by nosology differs significantly from the data of the Russian Cancer Registry (we used data for 2024 as an example) [25].

Compared with other studies, the highest overall mutation yield was observed in the study by Fiala et al. (18%) when analyzing only solid tumors, and in the study by Wang et al. (16.2%), where hematologic malignancies accounted for 17% (Figure 9). The difference in the overall mutation yield between the prospective and retrospective cohorts (27% vs. 13%) in our study is explained primarily by the significant difference in the percentage of hematologic neoplasms (3.5% vs. 32.6%, respectively). However, it can also be expected that patients with CPS have a more severe course of the disease, and their proportion among cancer survivors may slightly decrease with prolonged remission [7].

The diagnostic yield of genetic testing can also be affected by the panel size. Most studies have used a combination of methods: multigene panels of varying sizes together with WES and/or WGS. However, it should be noted that the use of a small panel (22 genes) resulted in the lowest mutation yield, 3.8% [31].

To date, approximately 200 genes associated with hereditary tumor syndromes have been described in the literature [32,33]. Genetic testing panels for hereditary cancer are mainly developed based on the NCCN guidelines for adult malignancies such as breast, ovarian, and prostate cancer [34,35], while no single list of genes has been developed for childhood cancer. Various criteria are used to select candidate genes, such as (1) relevance to childhood cancer, confirmed by the presence of a pathogenic variant in the gene in at least five patients with childhood cancer, and (2) the presence of a causal relationship between variants in the gene and cancer development. These criteria allowed the selection of 138 target genes [8]. Another study examined a research panel of 143 genes for genetic testing of pediatric CPSs [24]. 

In our study, we did not detect a significant difference in the diagnostic yield of genetic variants between the 415-gene panel and the clinical exome. This may be because the 415-gene panel included a fairly broad range of cancer-associated genes, and thus, the sets of targeted genes in these two panels overlapped considerably. Our study identified variants in 42 genes, including 16 genes associated with adult-onset syndromes. Although a link between mutations in adult-onset CPS genes and childhood cancer risk has not been proven [20], awareness of high-risk mutations within the family may be important for older relatives. Overall, testing using a broader gene panel (200 or more genes) should reduce the likelihood of missing a child with rare CPS, especially as universal genetic sequencing is increasingly being introduced into clinical practice [36,37]. 

However, targeted gene panel sequencing has evident limitations, as it does not detect usually large deletions/insertions or chromosomal rearrangements. For example, we did not detect a pathogenic variant in the *TSC1/2* genes in a patient with clinical features of tuberous sclerosis (subependymal giant cell astrocytoma, angiofibromas, and renal angiolipomas). We also did not identify a genetic defect in the *RB1* gene in a patient with bilateral retinoblastoma, despite the fact that in such cases the presence of a germline mutation of *RB1* is almost always assumed [38,39].

The *RB1* gene is an example of a target gene with a high probability of being associated with the disease. Therefore, Sanger sequencing and deep next-generation sequencing of the entire *RB1* gene are the main methods for identifying single nucleotide substitutions and small insertions/deletions, which comprise 70–75% of pathogenic *RB1* variants [40,41]. In addition, NGS together with allele-specific PCR (AS-PCR) are used for detecting low-level mosaicism. Deletions or duplications at the exon level, which occur in 15–20% of retinoblastoma cases, can be detected by multiplex ligation-dependent probe amplification (MLPA) [41,42], while array comparative genomic hybridization (aCGH) or karyotyping is applied to analyze large deletions affecting chromosome 13q14 [43]. 

A similar approach is applicable to the diagnosis of neurofibromatosis type 1 (NF1), which is one of the most common tumor syndromes with population frequency 1:3000 [17]. In our study, mutations in the *NF1* gene were detected in 36 patients, and 35/36 (97%) had pronounced phenotypic manifestations, enabling the clinical suspicion of this syndrome. However, it should be remembered that characteristic skin lesions in the form of café-au-lait spots are also found in other syndromes, for example, Legius syndrome (*SPRED1*), Noonan syndrome (*PTPN11*, *LZTR1*, *SOS1*), and LEOPARD syndrome (*PTPN11*, *RAF1*, *BRAF*), which necessitates the analysis of several genes [44]. In our study, a patient with astrocytoma and café-au-lait spots was clinically diagnosed with NF1, but sequencing results revealed a homozygous *PMS2* mutation associated with CMMRD. 

In cases where clinical features suggest a genetic defect in a specific gene, especially with well-known hot spots, Sanger sequencing is often used as a first-line genetic test. For instance, medullary thyroid cancer is associated with mutations in the *RET* gene, which are predominantly located in certain exons [45]. In Li–Fraumeni syndrome, up to 77% of mutations in the *TP53* gene are located between codons 125 and 300, corresponding mainly to the DNA-binding domain [46]. In our study, all identified *TP53* mutations were within these boundaries. The *VHL* gene, associated with von Hippel–Lindau syndrome, consists of three exons, making it feasible to analyze the entire coding sequence by Sanger sequencing [47].

Because deletions affecting individual exons or entire loci are characteristic of a number of CPS genes, for example, *NF1* (frequency 5–11%) [17], *TSC1* (frequency 8.5%) [48], and *WT1* (frequency 9.3%) [49], MLPA is typically used as the next step in genetic testing when gene sequencing results are negative. Epigenetic abnormalities can also make a significant contribution to the development of childhood neoplasms; for example, in nephroblastoma, methylation abnormalities associated with Beckwith–Wiedemann syndrome that promote tumor development are detected with a frequency of 3 to 16%, which underscores the relevance of methylation-specific MLPA [27,50]. 

Thus, for the most complete identification of genetic abnormalities predisposing to cancer, a comprehensive approach based on an understanding of the molecular background of the disease and the spectrum of mutational events is often necessary. Furthermore, panel sequencing is distinguished by its versatility allowing a single investigation to replace numerous individual tests for differential diagnosis of syndromes with overlapping phenotypic features. It should be noted that the applicability of NGS methods is rapidly expanding. Although MLPA remains the gold standard for identifying large rearrangements, it has now become possible to identify CNVs from NGS data using bioinformatics methods [51].

### 3.2. Causative Genes of Cancer Predisposition Syndromes in Childhood

In summary, we identified 126/886 (14.2%) PLP variants that were related to known CPSs with a high risk of developing malignancies. The greatest number of causative variants were found in the *NF1* and *TP53* genes. 

Mutations in *NF1* (36/886, 4% in the total sample) were most often associated with CNS tumors (21/36, 58%), primarily with OPG; as well as with tumors of the peripheral nervous system (PNST) (9/36, 25%), which corresponds to the known phenotype of neurofibromatosis type 1 [52]. In addition, rarer phenotypes were noted: nephroblastoma, pheochromocytoma and renal carcinoma in *NF1* mutation carriers [53,54,55]. Additionally, two cases of acute lymphoblastic leukemia (ALL) with *NF1* variants were found, while previously, *NF1* mutations were described only in patients with juvenile myelomonocytic leukemia [56]. Both in a previously reported case and in our study, AT/RT was detected in a patient with an *NF1* mutation and a characteristic phenotype; however, the causal relationship between the mutation and tumor development remains unclear [57].

The high frequency of *TP53* mutations (17/886, 2%) confirms the importance of excluding Li–Fraumeni syndrome in children with various tumors, primarily in patients with sarcomas. In our sample, the highest frequency of *TP53* mutations was observed in the patients with rhabdomyosarcoma (6/61, 10%) and osteosarcoma (8/107, 7.5%). According to the literature, the frequency of germline mutations in the *TP53* gene in soft tissue sarcomas is 12.8–28.1%, and in osteosarcomas 0.7–13.4%, respectively [58,59].

Mutations in the *DICER1* gene (12/886, 1.3%) were most frequently associated with thyroid neoplasms (6/85, 7%) and ovarian tumors (SCST) (3/14, 21%), as well as eRMS (2/14, 14%), all of which are part of the *DICER1* spectrum of neoplasms [60,61]. PLP variants in the *WT1* (*n* = 9), *TRIM28* (*n* = 4), and *REST* (*n* = 2) genes associated with nephroblastoma have been described in detail previously [27].

Mutations in the *PTEN* gene were detected in 10 patients, all of them had phenotypic features of Cowden syndrome: macrosomia and macrocephaly. 75% of patients (*n* = 6) had typical benign neoplasms (fibroadenomas, lipomas, thyroid adenomas), while 25% (*n* = 3) developed malignant tumors at an early age: pelvic germ cell tumor (4 years) and ovarian embryonal carcinoma (8 years). Germ cell tumors in patients with Cowden syndrome at an early age have been described previously, raising the question of including this phenotype in patient screening programs [62,63].

The *SDHB* gene encodes a subunit of the succinate dehydrogenase (SDH) complex (mitochondrial complex II) and is thus involved in the conversion of succinate to fumarate in the tricarboxylic acid cycle. Germline mutations of *SDHx*, including *SDHB*, result in loss of function and the development of various types of tumors, primarily pheochromocytoma and paraganglioma [64]. *SDHx* mutations have also been shown to be associated with gastrointestinal mesenchymal stromal tumor (GIST), renal cell carcinoma (RCC), pituitary adenomas; and rare phenotypes (thyroid cancer, neuroblastoma, and breast tumors) have been described [65,66]. In our study, mutations in the *SDHB* gene were identified in 7/886 (0.7%) patients with paragangliomas (*n* = 2), succinate dehydrogenase-deficient renal cell carcinoma (*n* = 2) and GIST (*n* = 2), but a rare combination of osteosarcoma as the first tumor and hormonally inactive pheochromocytoma as the second tumor was also detected in a patient with an *SDHB* mutation.

Rhabdoid tumor predisposition syndrome (mutations in the *SMARCB1* and *SMARCA4* genes) is characterized by an increased risk of developing malignancies called rhabdoid tumors (RT). These are rare, aggressive tumors, usually diagnosed in infants [67]; however, late onset and incomplete penetrance are also possible [68]. In our study, rhabdoid tumors of various localizations were detected in a total of 17 patients. Of these, the majority of patients (*n* = 12) had AT/RT of the brain, with ages ranging from 1 year to 12 years, and in three cases, mutations in the *SMARCB1* gene were detected (3/12, 25%), which is generally consistent with the literature data [69,70]. In all our patients with extracranial RT (kidneys, liver, soft tissues), tumors were diagnosed within the first year of life, and no mutations were detected. 

In one patient with osteosarcoma, we identified a nonsense mutation c.2542G>T (p.Glu848Ter) in the *SMARCAL1* gene, resulting in truncation of the protein. The SMARCAL1 protein, like SMARCB1 and SMARCA4, is a part of the SWI/SNF chromatin remodeling complex. Mutations in the *SMARCAL1* gene have been described in patients with osteosarcomas, and loss of heterozygosity in tumors has been demonstrated for loss-of-function mutations. Therefore, the *SMARCAL1* gene is considered a novel osteosarcoma predisposition gene [71,72].

Mutations in the *APC* gene (*n* = 3) in our study were associated with thyroid carcinoma. Two cases were papillary thyroid carcinoma, while one case presented with a rare histological subtype of papillary carcinoma—cribriform-morular tumor. In most cases, this tumor type is caused by a mutation in the *APC* gene and is the first manifestation of familial adenomatosis coli (FAP) [73,74].

Other CPS genes with matching phenotypes in our study were *EXT1*, *NBN*, *NF2*, *PDGFRB*, *PHOX2B*, *PMS2*, *PRKAR1A*, *PTCH1*, *PTPN11*, *RB1*, *RET*, *SUFU*, and *VHL*. A rare tumor type, ganglioneuroma, was detected in a patient with Noonan syndrome and a *PTPN11* mutation; such a case was previously described in an adult patient with Noonan syndrome [75]. 

The germline mutation p.Arg206His in the *ACVR1* gene found in three of our patients occurs in 95–97% of fibrodysplasia ossificans progressiva (FOP) cases, a rare autosomal dominant disorder characterized by genetically driven heterotopic ossification, which can mimic tumor growth but does not increase the risk of developing cancer [76]. At the same time, somatic *ACVR1* mutations were found in 20–25% of pediatric diffuse intrinsic pontine gliomas, a fatal brainstem tumor with a median survival of 8–10 months and no effective therapies [76,77].

### 3.3. Other Cancer Predisposition Syndrome Genes

Heterozygous pathogenic variants in genes of autosomal dominant syndromes with adult-onset manifestation (*BRCA1*, *BRCA2*, *BARD1*, *CHEK2*, *MSH2*, *MSH3*, *MSH6*, *PALB2*, *POLE*, and *RAD51C*) or in genes of autosomal recessive syndromes (*ATM*, *BLM*, *MSH3*, *MUTYH*, and *RECQL4*) were detected in 6.3% of patients. All these genes are involved in DNA repair processes associated with homologous recombination (*ATM*, *BARD1*, *BLM*, *BRCA1*, *BRCA2*, *CHEK2*, *PALB2*, *POLE*, *RAD51C*, and *RECQL4*) or mismatch repair (*MSH2*, *MSH3*, *MSH6*, and *MUTYH*).

In a recent study of pediatric patients with neoplasms, 3% of tumors were found to harbor pathogenic variants affecting one or more adult-onset syndrome genes, with some cases exhibiting loss of heterozygosity or a DNA mutational signature consistent with homologous recombination defects [22]. A meta-analysis of 11 studies that included comprehensive testing of germline variants in children and adolescents with cancer found statistically significant associations with the risk of developing brain tumors and other solid tumors for pathogenic variants in the *BRCA1/2* genes and mismatch repair genes [78], but no changes in surveillance are recommended due to low penetrance of pathogenic variants in adult-onset syndrome genes in childhood [20]. 

The identification of such mutations may initiate further testing and surveillance of close relatives, who may have an increased risk of developing cancer if they carry the mutation. In two our patients with fibroadenomas, mutations in the *BRCA1* and *BRCA2* genes were found. Although fibroadenoma is a benign tumor that becomes malignant in only 0.5–1% of cases, the presence of *BRCA1/2* mutations significantly increases this risk of developing cancer. Furthermore, the adolescent with fibroadenoma and *BRCA1* mutation had a family history of breast cancer, which may raise the question of earlier cancer surveillance in that concrete case.

In our study, clinically significant variants were detected in two or even three genes simultaneously in 6/176 (3.4%) patients. One variant usually was in a causative gene, while the second and third variants were identified in genes associated with adult-onset syndromes (e.g., *ATM, BRCA1*, *BRCA2*, *CHEK2*, *PALB2*, and *MUTYH*), but in all these cases the phenotype corresponded to the mutation in the primary gene. This clearly indicates the absence of a causal relationship between mutations in adult-onset genes and tumor development. However, it is assumed that additional mutations may have a modifying effect and influence the penetrance and clinical course of disease, as well as the combination of two or more alleles of different adult-onset genes in one genotype [79].

The vast majority of syndromes we identified (164/176, 93%) are inherited in an autosomal dominant manner. However, 2.3% (4/176) of cases were homozygous for alleles of autosomal recessive syndromes (CMMRD syndrome, Nijmegen syndrome), a finding that requires special attention in genetic counseling for families. Nijmegen syndrome in both cases was associated with a mutation c.657_661del5 in the *NBN* gene, which has a founder effect in European populations of Slavic origin [80], where this allele occurs with a population frequency of about 0.003.

The presence of monoallelic variants of the *MUTYH* and *BLM* genes associated with autosomal recessive syndromes is considered carrier status in most studies, since their overall frequency in the population is quite high. Homozygous mutations in the *RECQL4* gene cause Rothmund–Thomson syndrome, which is characterized by an increased risk of developing bone sarcomas. However, no risk of cancer was described for heterozygous carriers in families of patients with this syndrome [81]. But, recent study of pediatric patients with osteosarcomas revealed an increased frequency of heterozygous loss-of-function variants in the *RECQL4* gene [82], that may indicate their potential role in the development of this tumor. In our study, heterozygous variants of the *RECQL4* gene were detected only in patients with osteosarcomas.

Two patients with nephroblastoma and RCC had the same mutation, *BARD1* c.2300_2301del, which was previously described in Russian patients with breast cancer [83]. Germline mutations in the *BARD1* gene have been previously described in patients with renal neoplasms [84]. The *BARD1* gene is involved in homologous recombination, and the formation of the BRCA1-BARD1 complex is critical for the repair of double-strand DNA breaks and the maintenance of genomic stability. But, genetic profiling of the tumor tissue from the patient with RCC did not reveal the characteristic deficiency of homologous recombination, that did not support its role in the tumor development. 

### 3.4. Risk of Second Malignant Tumor in Pediatric Patients with Cancer

Subsequent malignant neoplasms (SMNs) are the leading cause of premature death in childhood cancer survivors [85]. In most cases, these are solid tumors associated with radiation, as well as the use of alkylating agents, platinum compounds, and anthracyclines [86]. One of the common secondary tumors is follicular or papillary thyroid carcinoma, which most often develops in patients with leukemia, non-Hodgkin lymphoma, and renal cancer [87]. Pathogenic/likely pathogenic variants in CPS genes are associated with an increased risk of developing SMNs; it has been shown that childhood cancer survivors with PLP mutations in CPS genes were 4.3 times more likely to develop a secondary tumor compared to those who did not have these mutations. The highest risk was noted for carriers of mutations in the *TP53* and *RB1* genes [88].

In our study, SMNs were detected in 3.5% of patients (*n* = 31); the most common second tumor was thyroid carcinoma—45% (14/31). The frequency of second tumors in the group of patients with CPS mutations was significantly higher compared to patients without mutations: 8.5% (15/176) vs. 2.3% (16/710); OR, 4.0; 95% CI: 2.0–8.3; *p* = 0.0003. Moreover, in the group of patients without CPS mutations, thyroid carcinoma developed in two patients with ALL, which is probably attributable to previous treatment. Among all patients with *TP53* mutations, second tumors developed in 24% (4/17) over a period of 10 to 15 years, and among patients with *RB1* mutations, second tumors arose in 50% (2/4) of cases. It should be noted that mutations in the *CHEK2* and *RAD51C* genes, associated with homologous recombination deficiency, could be a risk factor for the development of a second tumor, thyroid carcinoma, as a complication of treatment [89].

### 3.5. Genotype-Driven and Phenotype-Driven Approaches: Lessons from the Study

To identify patients with germline mutations based on the Jongmans phenotypic criteria, we examined two multivariable logistic regression models. Both models demonstrated good discriminatory ability, but the model with a quantitative indicator (Total score) consistently outperformed the binary model both in terms of AUC (0.886 vs. 0.849 in cross-validation) and AIC (ΔAIC = 54.17). This confirms that the severity of the phenotype is a more informative predictor than the presence of the criteria themselves. Tumor type (bone sarcomas and soft tissue tumors) was also an independent predictor of the presence of a CPS mutation in both models. 

It is important to note that all four Jongmans criteria are clinical manifestations of hereditary tumor syndromes. Thus, we are predicting the cause from its effects; however, this does not reduce the clinical value of the criteria in patient selection for genetic testing. The high values obtained (OR = 24.31 in the binary model and OR = 10.69 per criterion in the quantitative model) should be viewed as a reflection of a close genotype–phenotype association, rather than as proof of a causal relationship. All estimates of the discriminatory ability of the models (sensitivity, specificity, NPV, and PPV) were obtained based on the results of 10-fold cross-validation of the original sample. Since external validation on an independent cohort was not conducted, we consider these results preliminary and do not exclude the possibility of further re-evaluation of the predictive performance of the models. 

In our study, 312 of 886 patients (35%) in the total sample met Jongmans’ criteria. Among them, 113 patients were identified with a mutation in the known CPS gene. Thus, the use of a phenotype-driven approach allowed us to increase the diagnostic yield from 14.2% in the total sample to 36% in the Jongmans-positive group of patients, identifying approximately 90% (113/126) of all causative mutations. However, in 10% (13/126) of patients, the presence of mutations could not be suspected based on phenotype assessment alone. Remarkably, in this group of patients, clinically significant mutations in the *TP53* gene were detected in six patients (46%); among whom three had eRMS, and the other three had bone tumors (osteosarcomas and chondrosarcoma). Mutations in the *TP53* gene are associated with an extremely high lifetime risk of developing cancer (70% in men and up to 100% in women) [58]. In addition, patients with mutations in such causative genes as *WT1*, *DICER1*, *SMARCAL1*, *PHOX2B*, and *SMARCA4* (which was associated with a non-matched phenotype in our study), were not included in the group for subsequent genetic testing. This may be due not only to an incomplete phenotype assessment, since we did not take into account the toxicity criterion, but also to the variability of clinical manifestations and incomplete penetrance of the CPS genes.

Clinical selection-based genetic testing using the MIPOGG tool [11] and phenotype-agnostic germline sequencing were compared previously. Only one (4%) of the 27 causative CPSs was missed by the phenotype-driven approach and was identified solely by phenotype-agnostic CPS gene sequencing, and it was *TP53* variant de novo [24]. This led the authors to conclude that the phenotype-driven approach should be prioritized in clinical practice when testing children with cancer. Further development of the phenotype-driven approach may be associated with improved algorithms for phenotypic assessment of patients and selection criteria [90], which will reduce the proportion of false-negative results.

As has been demonstrated in many previous studies, genetic testing based on a phenotype-driven approach is an effective method for diagnosing most hereditary cancer syndromes [2,9,24]. On the other hand, the increasing availability of NGS methods is driving the transition toward screening all children with cancer, an approach that is becoming more relevant for identifying actionable alterations in tumor tissue [91]. An example of large-scale tumor and germline testing is the Pediatric MATCH study, which examined actionable tumor alterations in 1000 patients and compared them with treatment outcomes [92], or the Genomes for Kids study, which included whole-genome sequencing (WGS), whole-exome sequencing (WES), and RNA sequencing (RNA-seq) of paired tumor and germline samples from 309 prospectively identified children with cancer, unselected for tumor type [18]. Universal testing of pediatric cancer patients is expected to increase the identification of cancer predisposition syndromes in children, as well as facilitate the discovery of new associations between genetic alterations and tumor development and the identification of targets for therapy.

### 3.6. Limitations of the Study

The study has several limitations. First, the study cohort is predominated by solid tumors (83%), which are much more frequently associated with hereditary cancer predisposition syndromes than hematologic malignancies. The oncologists were more likely to refer for genetic counseling patients with certain tumor types or with suspected cancer syndromes based on phenotypic features. Thus, in total sample, CNS tumors, renal tumors, bone tumors, and soft-tissue tumors were overrepresented. Therefore, the frequency of mutations in cancer-associated genes in our study may be higher than that in the general pediatric cancer population, and more likely reflects the characteristics of the selected cohort, which is dominated by tumors known to have a higher probability of underlying genetic predisposition. Second, genetic testing was performed only using NGS methods. Panel and clinical exome sequencing are designed to detect point mutations in the coding regions of target genes and do not allow for the identification of deep intronic variants, large insertions/deletions, chromosomal rearrangements, or aberrant methylation patterns. Consequently, the mutational landscape in the study may be incomplete and some percentage of patients with CPS were not identified. Third, the samples of close relatives were not available in all cases. Segregation analysis plays an important role in assessing the clinical significance and penetrance of the identified variants. Forth, when designing the multivariable regression models, the estimates of discriminatory ability (AUC) were obtained by conducting 10-fold cross-validation and were not externally validated, which may lead to an overestimation of predictive values. Therefore, the obtained results should be considered preliminary, and external validation on independent cohorts is required to confirm their clinical applicability. Finally, our study focused on the detection of germline mutations, while analysis of tumor tissue could provide additional insights into the contribution of the identified mutations to disease pathogenesis and cancer development.

## 4. Materials and Methods

### 4.1. Patients

The study included 886 patients aged 0 to 18 years with lymphoproliferative and solid neoplasms of various localizations. Patient enrollment and genetic testing were conducted between 2018 and 2025 (7 years). Of these, 477 patients were treated at the N.N. Blokhin Russian Cancer Research Center of the Ministry of Health of the Russian Federation (prospective cohort), and 409 patients were undergoing rehabilitation at the Rehabilitation Research Center “Russkoye Pole” of the Dmitry Rogachev National Medical Research Center of Pediatric Hematology, Oncology, and Immunology (retrospective cohort). For all patients, the following data were collected: clinical diagnosis, histological tumor type, family history, presence of other tumors, and phenotype description. All patients underwent genetic counseling. The phenotype was assessed retrospectively by review of medical records, and fulfillment of the Jongmans criteria (positive family history, multiple tumors, rare or high-risk tumors, presence of congenital anomalies) was registered (Table S2). All 886 patients underwent genetic testing using next-generation sequencing (NGS) with the following panels: TruSight One Expanded (Illumina, 6794 genes, *n* = 390) or a custom panel (Roche, 415 cancer-associated genes, *n* = 496). Genomic DNA was isolated from peripheral blood lymphocytes using the HiPure Blood DNA Mini Kit (Magen Biotechnology Co., Guangzhou, China) according to the manufacturer’s protocol. Before genetic testing, all patients signed an informed consent form.

### 4.2. Next-Generation Sequencing

Library preparation was performed using a hybridization-based selective enrichment method with biotinylated probes targeting the coding regions of the target genes. The TruSight One Expanded Sequencing Panel (Illumina, San Diego, CA, USA) covers the coding regions of 6794 genes (the full gene list is available on the Illumina website: https://support.illumina.com/downloads/trusight_one_sequencing_panel_product_file.html, accessed on 15 May 2026). The Illumina DNA Prep with Enrichment kit (Illumina, San Diego, CA, USA) was used for library preparation and hybridization. The custom panel was designed using the HyperDesign online service (Roche, Basel, Switzerland) and includes the coding regions of 415 cancer-associated genes [23]. The KAPA HyperPrep Kit (Roche, Basel, Switzerland) was used to prepare the libraries, and hybridization was performed using the KAPA HyperCap Workflow v3.0 kit (Roche, Basel, Switzerland). Sequencing was performed on a NextSeq2000 instrument (Illumina, San Diego, CA, USA) using the P1 300-cycle kit (Illumina, San Diego, CA, USA). The average coverage was 150× for The TruSight One Expanded Sequencing Panel and 250× for 415 cancer-associated genes panel, the percentage of the target region with at least 50× coverage was ≥90%, and the quality score was ≥99.9%.

### 4.3. Bioinformatic Analysis

Sequencing quality was assessed using FastQC. Read pairs were aligned to the reference genome (hg38) using the BWA-MEM2 algorithm. Duplicate sequences were then identified and removed using Picard MarkDuplicates. Base quality scores were recalibrated, and genetic variants were identified using Genome Analysis Toolkit (GATK) tools, specifically BQSR for score recalibration and HaplotypeCaller for variant calling.

Filtering was based on total read depth (DP) ≥ 20×, quality score (QUAL) ≥ 30, and alternative allele depth (AD) ≥ 5. For heterozygous variants, a variant allele frequency (VAF) threshold of ≥30% was applied, and for homozygous variants, VAF ≥ 90% was required. To minimize false-positive calls, standard GATK hard filters (QD < 2.0, FS > 60.0, MQ < 40.0) were applied, and variants located in low-complexity regions or homopolymer stretches were excluded. For population frequency filtering, we applied the ACMG BA1 criterion: variants with a global allele frequency > 0.001 (0.1%) in any gnomAD population were excluded for autosomal dominant genes, and >0.005 (0.5%) for autosomal recessive genes. For genes with well-established pathogenic founder variants (e.g., *CHEK2*, *MUTYH*), the BA1 criterion was not applied, and such variants were retained if they had consistent pathogenic annotations in ClinVar or the literature.

### 4.4. Variant Classification

Variant classification was performed according to the ACMG/AMP 2015 guidelines for the interpretation of sequence variants [93]. We applied a semi-automated approach in which criteria that could be objectively evaluated using computational tools were assigned automatically, while criteria requiring clinical or familial information were curated manually. Automated evidence codes were obtained using three independent variant interpretation platforms Franklin (Genoox) accessed on 8 July 2026. This tool provided systematic assessments of population allele frequencies based on gnomAD and the 1000 Genomes Project, enabling the application of BA1, BS1, and PM2, as well as computational predictions from multiple in silico algorithms such as SIFT, PolyPhen-2, CADD, REVEL, MetaRNN, and AlphaMissense, which supported PP3 or BP4. Transcript-level annotations from RefSeq and Ensembl were used to assess variant consequence and loss-of-function effects, contributing to PVS1, PM4, and BP3, while cross-referencing with ClinVar, HGMD, and PubMed provided literature and database evidence for PS1, PM5, and PP5, the latter being used with caution and only when primary evidence was unavailable. All automated classifications were reviewed and, when necessary, overridden based on additional clinical data. 

The following criteria were added manually according to ClinGen guidelines (https://www.clinicalgenome.org/, accessed on 10 July 2026): PS2 or PM6 for confirmed or assumed de novo occurrence, PP4 when the patient phenotype was highly specific to the disease associated with the gene, and PP1 for segregation of the variant with disease in affected family members. Finally, the overall evidence for each variant was summarized using a quantitative scoring system, where each criterion was assigned as a weight depending on its strength: Very Strong (±8), Strong (±4), Moderate (±2), or Supporting (±1). The total score was calculated as the sum of pathogenic and benign evidence, and the final classification was determined as Pathogenic (≥10 points), Likely Pathogenic (6–9 points), VUS (0–5 points), Likely Benign (−2 to −6 points), or Benign (≤−7 points) [93,94]. All discordant or borderline cases were discussed by a multidisciplinary team to ensure the clinical validity of the final interpretation. The complete set of ACMG evidence codes with their strength levels assigned to each variant is provided in the Supplementary Material (Table S3).

### 4.5. Sanger Sequencing

Any variant classified as pathogenic or likely pathogenic was verified by Sanger sequencing. When DNA of family members was available, segregation analysis was performed. Primers were designed using Primer-BLAST (https://www.ncbi.nlm.nih.gov/tools/primer-blast/, accessed on 25 March 2026), which utilizes Primer3 software (version 2.5.0) [95]. The PCR mixture (final volume 25 μL) consisted of 1× PCR-Buffer-B for Taq DNA polymerase; 2 mM MgCl_2_; 1 unit SynTaq DNA polymerase; 0.2 mM dNTPs; 0.04 μM of forward and reverse primers; and 20–30 ng DNA. All PCR reagents were produced by SYNTOL (Moscow, Russia), and primers were synthesized by Evrogen (Moscow, Russia). PCR was performed on a T100 Bio-Rad thermocycler (Hercules, CA, USA). The PCR cycling conditions were as follows: 3 min at 95 °C, followed by 35 cycles of 30 s at 95 °C, 30 s at 60 °C, 30 s at 72 °C, and a final extension at 72 °C for 2 min. Sanger sequencing was performed using a 3500 Series Genetic Analyzer (Applied Biosystems, Thermo Fisher Scientific, Waltham, MA, USA). 

### 4.6. Statistical Analysis

The 2 × 2 contingency tables and Fisher’s exact test were used for statistical examination. Odds ratios (OR) for associations were determined and the 95% confidence intervals (95% CI) were calculated. Statistical analysis was performed using the GraphPad InStat 3 (GraphPad Software Inc., La Jolla, CA, USA), and *p*-value < 0.05 was considered significant. 

Multivariable logistic regression was performed using Python (version 3.x) with the pandas, numpy, and matplotlib packages. The statsmodels package was used to construct a logistic regression model and check for multicollinearity. The sklearn package was used to assess model quality. To analyze the association between phenotypic traits (Jongmans criteria) and the presence of genetic mutations, two models were constructed. Both models adjusted for potential confounders: gender, age at diagnosis, tumor type, and cohort (PC—prospective, RC—retrospective). CNS tumors were chosen as the reference group for tumor type, as they are the most numerous and clinically homogeneous group. Model 1 included a binary predictor—the presence/absence of Jongmans criteria (0 = no, 1 = yes). Model 2 included a quantitative predictor—the total score (0–4).

To test for multicollinearity among predictors, the Variance Inflation Factor (VIF) was calculated for all independent variables. For the categorical variable “tumor type,” VIF was calculated separately for each dummy variable, as this approach allows assessment of individual contributions and detection of potential collinearity within the group. VIF values below 5 were considered acceptable, indicating the absence of significant multicollinearity. Results are presented as odds ratios (OR) with 95% confidence intervals (CI) and p values. Results with *p* < 0.05 were considered statistically significant. To assess the discriminatory power of the models, ROC-AUC analysis was performed on the entire sample (apparent AUC). To evaluate the predictive ability of the models, 10-fold cross-validation was conducted. To assess the quality of the models, metrics such as sensitivity, specificity, positive predictive value (PPV), and negative predictive value (NPV) were also calculated. Graphical visualization of the results was performed using forest plots to display OR values and confidence intervals for all predictors (Supplementary, Table S9).

## 5. Conclusions

In this study, multigene panel or clinical exome sequencing of 886 Russian pediatric and adolescent patients with a wide spectrum of neoplasms revealed pathogenic or likely pathogenic germline variants in cancer-associated genes in 20.5% of cases. Variants causative for well-known pediatric cancer predisposition syndromes accounted for 14.2% of all patients, which is at the higher limit of previously reported studies. This high frequency is associated with the overrepresentation of solid tumors in the study population, but also underscores the clinical utility of broad genetic testing in this patient group.

The overall total mutation rate varied significantly among patient groups with different tumor types. The highest diagnostic yield was observed in retinoblastoma (80%), peripheral nerve sheath tumors (60%), and pheochromocytoma/paraganglioma (47%), while the lowest yield was found in neuroblastoma (12%) and hematologic malignancies (4.6%). These data provide a roadmap for prioritizing patients for genetic counseling and testing based on tumor type. Among the most frequently affected causative genes were *NF1*, *TP53*, *DICER1*, *WT1*, *PTEN*, and *SDHB*. Rare phenotypes identified in the study expand our understanding of tumorigenesis and may uncover potential drivers of cancer.

Pathogenic or likely pathogenic variants in genes typically associated with adult-onset cancer syndromes (e.g., *BRCA1*, *BRCA2*, mismatch repair genes) were detected in 6.3% of patients. Our study did not support the role of these genes in childhood cancer; nevertheless, detecting mutations in adult-onset syndrome genes may carry significant implications for family screening and long-term surveillance.

Patients with multiple primary tumors showed a significantly higher frequency of causative germline variants (42%) compared to those with a single tumor (13%, *p* < 0.0001), highlighting the importance of genetic testing in this subgroup. Multivariable logistic regression models demonstrated that phenotypic criteria are strong independent predictor of CPS genotype and their application allows to increase substantially the diagnostic yield among pediatric patients with neoplasms. The use of phenotypic assessment algorithms, particularly for children with hematologic malignancies, is important to reduce the need for potentially unnecessary genetic screening. At the same time, 10% of patients with proven cancer predisposition syndromes did not meet any of the Jongmans criteria examined, indicating that current clinical selection tools may miss a certain number of affected individuals, especially among those carrying deleterious mutations in the *TP53* gene.

Collectively, our findings support the utility of combining multigene panel sequencing with a phenotype-driven approach for pediatric patients with a wide spectrum of neoplasms. The inclusion of both childhood-onset and adult-onset cancer predisposition genes in multigene panel testing is justified, as it enables personalized treatment, surveillance for second malignancies, and cascade testing in at-risk relatives. For retinoblastoma and peripheral nerve sheath tumors, the standard approach, which appears more productive, is extensive genetic testing of the entire gene sequence (*RB1* or *NF1*, respectively) followed by copy number variation (CNV) analysis, and for medullary thyroid carcinoma Sanger sequencing of selected exons in the *RET* gene. At the current stage, broad germline sequencing is considered mainly as a research option to complement phenotype-driven approach and not to miss clinically important subgroups of patients. Future prospective studies with unselected populations and comprehensive genomic analyses (including copy number variant detection and epigenetic testing) will be essential to refine these recommendations further.

## Figures and Tables

**Table 1:** Clinical characteristics of patient cohorts (ALL—acute lymphoblastic leukemia, AML—acute myeloid leukemia, CNS—central nervous system, PNST—peripheral nerve sheath tumors).

Clinical Parameters		Prospective Cohort (*n* = 477)	Retrospective Cohort (*n* = 409)	Total Study (*n* = 886)
Sex	Male	184	207	391 (44%)
	Female	293	202	495 (56%)
Median age at diagnosis of first tumor (range), years 6 (from 0 to 18)				
Age at diagnosis, years (*n* = 886)	<5	175	177	352 (40%)
	5–9	93	123	216 (24%)
	10–14	121	90	211 (24%)
	≥15	88	19	107 (12%)
Patients with different number of tumors (*n* = 886)	One primary tumor	448	407	855 (96.6%)
	Two primary tumors	26	2	28 (3.1%)
	Three primary tumors	3	0	3 (0.3%)
**Total number of tumors**		509	411	920 (100%)
Tumor type	Hematologic neoplasms	18	134	152 (17%)
	ALL	6	96	102
	Hodgkin lymphoma	2	15	17
	Non-Hodgkin lymphoma	6	20	26
	Other	4	3	7
	CNS tumors	89	90	179 (19%)
	Astrocytoma	17	38	55
	Medulloblastoma	17	18	35
	Optic pathway glioma	14	5	19
	Other CNS tumors	41	29	70
	Peripheral nerve sheath tumors (PNST)	15	0	15 (1.6%)
	Bone tumors	23	104	127 (14.3%)
	Osteosarcoma	14	93	107
	Ewing sarcoma	3	11	14
	Other	6	0	6
	Soft-tissue tumors	67	17	84 (9%)
	Rhabdomyosarcoma	45	16	61
	Other soft-tissue tumors	22	1	23
	Renal tumors	84	37	122 (13.5%)
	Nephroblastoma	69	36	105
	Other	16	1	17
	Thyroid carcinoma/adenoma	83	2	85 (9%)
	Papillary carcinoma	56	2	58
	Follicular carcinoma	21	0	21
	Other	6	0	6
	Neuroblastoma/ganglioneuroma	28	22	50 (5.4%)
	Gonadal tumors	27	2	29 (3%)
	Sex cord stromal tumors	14	0	14
	Germ cell tumors	7	2	9
	Other	6	0	6
	Extra-gonadal germ cell tumors	6	2	8 (1%)
	Neuroendocrine tumors	15	0	15 (1.6%)
	Retinoblastoma	5	0	5 (0.6%)
	Breast fibroepithelial tumors	30	0	30 (3%)
	Other	19	0	19 (2%)

## Data Availability

Data is contained within the article or Supplementary Material.

## Supplementary Materials

The following supporting information can be downloaded at: https://www.mdpi.com/article/10.3390/ijms27146395/s1.
