# Supplementary material for: Clinical Impact of Germline Multigene Sequencing in Pediatric Cohorts with a Wide Spectrum of Neoplasms
Source: Int J Mol Sci. 2026 Jul 18;27(14):6395. doi: 10.3390/ijms27146395 (PMC13410190; doi:10.3390/ijms27146395)
Supplement: Supplementary file 1 [file ijms-27-06395-s001.zip › ijms-4377847-supplementary/Table S10.pdf]

Table S10. Frequency of PLP variants associated with hereditary tumor syndromes among pediatric patients in different studies. WES—whole exome sequencing, WGS—whole genome sequencing, CNS—central nervous system.

Study	Population	Type of study, method	Frequency of clinically significant variants
Zhang et al. 2015 [1]	1120 patients:<br>Leukemia/lymphoma—52.5%;<br>CNS tumors—21.9%;<br>Non-CNS tumors—25.6%	Retrospective,<br>WGS or WES	8.5% total<br>(CNS tumors 8.6%, non-CNS solid tumors 6.7%, leukemia 4.4%)
Parsons et al., 2016 [6]	150 patients:<br>CNS tumors—37%;<br>non-CNS tumors—63%	Prospective, tumor and germline WES	Germline WES:<br>10% total
Oberg et al., 2016 [29]	101 patients:<br>Hemoblastoses—36%;<br>Solid tumors—64%	Prospective, matched tumor–normal WES sequencing	14% total germline variants in CPS genes
Grobner et al., 2018 [30]	914 patients:<br>Leukemia/lymphoma—16%;<br>CNS tumors—58%;<br>Non-CNS tumors—28.7%	Retrospective and prospective tumor sequencing, WES, WGS (162 genes investigated for germline CPS)	7.6% total germline variants
Fiala et al., 2020 [3]	751 patients:<br>CNS tumors—19%;<br>Non-CNS tumors—81%	Prospective, matched tumor–normal sequencing of 468 genes, germline data analysis in 88 genes	18% total;<br>13% in moderate- and high-penetrance dominant genes
Wong et al., 2020 [5]	252 patients:<br>Leukemia/lymphoma—17%;<br>CNS tumors—37%;<br>Non-CNS tumors—46%	Prospective, tumor and germline WGS, high-risk pediatric cancer patients	16.2% total
Wagener et al., 2021 [4]	160 patients:<br>Leukemia/lymphoma—51.3%;<br>CNS tumors—20%;<br>Non-CNS tumors—28.7%	Trio WES	13.8% total
Stedingk et al., 2021 [31]	790 patients:<br>Leukemia/lymphoma—50%;<br>CNS tumors—19%;<br>Non-CNS tumors—31%	Retrospective,<br>target panel sequencing of 22 genes	3.8% total
Bakhuizen et al., 2023 [2]	824 patients:<br>Hemoblastoses—41%;<br>Solid tumors—59%	Retrospective, germline sequencing, different methods	8.6% total<br>(Hemoblastoses 5.4%, CNS tumors 7.8%, other solid tumors 12.6%)
Stoltze UK et al. 2025 [7]	1127 patients:<br>Leukemia/lymphoma—50%<br>CNS tumors—24%;<br>Non-CNS tumors—46%	Prospective and retrospective, WGS	16% total,<br>9% causative CPS variants in prospective cohort
Present study	886 patients:<br>Leukemia/lymphoma—17%<br>CNS tumors—19%;<br>Non-CNS tumors—64%	Prospective and retrospective, clinical exome (6794 genes), target panel (415 genes) sequencing	20.5% total, 14.2% causative CPS variants
