# Supplementary material for: Clinical Impact of Germline Multigene Sequencing in Pediatric Cohorts with a Wide Spectrum of Neoplasms
Source: Int J Mol Sci. 2026 Jul 18;27(14):6395. doi: 10.3390/ijms27146395 (PMC13410190; doi:10.3390/ijms27146395)
Supplement: Supplementary file 1 [file ijms-27-06395-s001.zip › ijms-4377847-supplementary/Table S2. Jongmans_MIPOGG criteria.pdf]

Table S2. Modified Jongmans Criteria and McGill Interactive Pediatric OncoGenetic Guidelines (MIPOGG)

I. Family history

- Known cancer predisposition syndrome
- Close relative (first or second degree) with cancer at ≤ 18 years OR a parent/sibling/half-sibling with cancer at ≤ 50 years
- Close relative (first or second degree) same cancer type or same organ affected by cancer at any age
- Close relative (first or second degree) with multiple primary tumors

II. Rare or "high risk" tumors

- Angiomyolipoma
- Atypical Teratoid Rhabdoid Tumor (ATRT)
- Cardiac rhamdomyoma
- Cerebellar gangliocytoma
- Choroid plexus carcinoma
- Cribriform-morular thyroid carcinoma
- Cystic nephroma
- Desmoid tumor
- Displastic cerebellar gangliocytoma
- Enlolymphatic sac tumor
- Extrarenal rhabdoid tumor
- Gardner fibroma Gastrointestinal stromal tumor (GIST)
- Hemangioblastoma
- Hepatoblastoma
- Juvenile Myelomonocytic Leukemia (JMML)
- Low hipoploid acute lymphoblastic leukemia
- Malignant Peripheral Nerve Sheath Tumor (MPNST)
- Medullary thyroid carcinoma
- Ovarian Sertoly-Leydig cell tumor
- Paraganglioma
- Parathyroid tumor
- Pheochromocytoma
- Pineoblastoma
- Pituitary blastoma
- Pleuropulmonary blastoma (PPB)
- Plexiform neurofibroma
- Renal angiomyolypoma
- Renal cell carcinoma
- Renal
- Retinoblastoma rhabdoid tumor
- Shwannoma
- Subependymal giant cell tumor
- Thichilemomma
- Adult cancer (colorectal, ovarian, breast, lung, cervix, uterus, bladder, basal cell carcinoma, etc)

III. Multiple primary tumors or bilateral and multifocal tumor

- > 1 primary tumor (asynchronous or synchronous)
- Bilateral or multifocal primary tumors

IV. Congenital malformations and other features

- Congenital anomalies (organs, bones, oral clefting, teeth, eyes, ears, brain, urogenital anomalies, etc.)
- Facial dysmorphisms
- Intellectual disability
- Aberrant growth (length, head circumference, birth weight, asymmetric growth)
- Skin anomalies (aberrant pigmentation i.e. > 2 café-au-lait spots, vascular skin changes, hypersensitivity for sunlight, multiple benign tumors of the skin)
- Hematological disorders (pancytopenia, anemia, thrombocytopenia, neutropenia)
- Immune deficiency
