# Supplementary material for: Clinical Impact of Germline Multigene Sequencing in Pediatric Cohorts with a Wide Spectrum of Neoplasms
Source: Int J Mol Sci. 2026 Jul 18;27(14):6395. doi: 10.3390/ijms27146395 (PMC13410190; doi:10.3390/ijms27146395)
Supplement: Supplementary file 1 [file ijms-27-06395-s001.zip › ijms-4377847-supplementary/Table S4. Patients with clinically significant variants in more than one gene.docx.pdf]

Table S4. Patients with clinically significant variants in more than one gene

#	Sample ID	Sex	Age	Genes	Phenotype
1	P682	F	0	*BRCA2* (Chr13:32339641 c.5286T>G , p.Tyr1762Ter)	Bilateral nephroblastoma
				BARD1 (Chr13:32339641 c.2300_2301del, p.Val767AspfsTer4)	
2	P264	M	1	*BRCA2* (Chr11:32417641 c.8606_8607del, p.Ile2869fs)	Nephroblastoma
				WT1 (Chr11:32417641 c.901C>T, p.Gln301Ter)	
3	P134	F	0	*CHEK2* (Chr19:58547713 c.1100delC, p.Thr367fs)	Bilateral nephroblastoma
				TRIM28 (Chr19:58547713 c.839+1G>A)	
4	R914	F	7	*BRCA1* (Chr17:43093844 c.1687C>T, p.Gln563Ter)	Pilocytic astrocytoma
				CHEK2 (Chr17:43093844 c.1100delC, p.Thr367fs)	
5	P488	M	15	*DICER1* (Chr14:95124441 c.1128_1132del, p.Lys376AsnfsTer11)	Follicular thyroid carcinoma
				MUTYH (Chr14:95124441 c.1103G>A, p.Gly368Asp)	
				PALB2 (Chr14:95124441 c.926_927del, p.Ile309LysfsTer2)	
6	P96	F	14	*CHEK2* (Chr22:28695869 c.1100delC, p.Thr367fs)	Multiply fibroadenomas
				PTEN (Chr10:87952263 c.634+4A>C)	
7	P121	F	13	*ATM* (Chr11:108251026-108251027 c.1564_1565del, p.Glu522fs)	Multiply fibroadenomas
				PTEN (Chr11:108251026-108251027 c.253+2T>G)	
8	P103	M	0	*BRCA1* (Chr17:43092245 c.3286C>T, p.Gln1096Ter)	Infantile myofibromatosis
				MUTYH (Chr17:43092245 c.736C>T, p.Arg246Trp)	
				PDGFRB (Chr17:43092245 c.1706T>C, p.Ile596Thr)	
