# Supplementary material for: Clinical Impact of Germline Multigene Sequencing in Pediatric Cohorts with a Wide Spectrum of Neoplasms
Source: Int J Mol Sci. 2026 Jul 18;27(14):6395. doi: 10.3390/ijms27146395 (PMC13410190; doi:10.3390/ijms27146395)
Supplement: Supplementary file 1 [file ijms-27-06395-s001.zip › ijms-4377847-supplementary/Table S5. Distribution of pathogenic genotypes among different tumor types.pdf]

Table S5. Distribution of pathogenic genotypes among different tumor types.

Tumor type	Genes of hereditary cancer syndromes		No cancer risk
	Matching phenotype	Non-matching phenotype	
Hematologic neoplasms (n = 152)	*NF1 (2)*	*BRCA2 (1), MSH2 (1)*<br>*BLM (1) CHEK2 (1), MUTYH (1)*	
PLPV (total) = 7/152 (4,6%)	2/152 (1,3%)	5/152 (3,3%)	
CNS (n = 179)	*NF1 (20), SMARCB1 (3), TP53 (2), NF2 (1), PTCH1 (1), SUFU (1), NBN*hom *(1), PMS2*hom *(1)*	*BRCA1 (1), BRCA2 (1), MSH2 (1), DDX41 (1), PALB2 (1), CHEK2 (4), MUTYH (2)*	
PLPV (total) = 41/179 (23%)	30/179 (17%)	11/179 (6%)	
PNST (n = 15)	*NF1 (9)*	0	
PLPV (total) = 9/15 (60%)	9/15 (60%)	0	
Osteosarcoma (n = 107)	*TP53 (8), SMARCAL1 (1), SDHB (1), RB1 (1)*	*BRCA1 (2), RECQL4 (2), SMARCA4 (1), ATM (1), MSH3 (1), RAD51C (1)*	
PLPV (total) = 19/107 (19%)	11/107 (11%)	8/107 (8%)	
Other bone tumors (n = 20)	*TP53 (1), EXT1 (2)*	*MSH6 (1), CHEK2 (1)*	
PLPV (total) = 5/20 (25%)	3/20 (15%)	2/20 (10%)	
Rhabdomyosarcoma (n = 61)	*TP53 (6), NF1 (3), NBN* hom *(1), DICER1 (2), PMS2* hom *(1), RB1 (1)*	*MSH6 (1)*	
PLPV (total) = 15/61 (25%)	14/61 (23%)	1/61% (2%)	
Other soft tissue tumors (n = 23)	*PTEN (2), PRKAR1A (2), SDHB (2), DICER1 (1), PDGFRB (1)*	*BRCA1 (1), MUTYH (1)*	*PKHD1 (1)*
PLPV (total) = 11/23 (48%)	8/23 (35%)	3/23 (13%)	1/23 (4%)
Nephroblastoma (n = 105)	*WT1 (9), TRIM28 (4), REST (2), TP53 (1), NF1 (1)*	*BARD1 (1), BRCA2 (2), POLE (1), CHEK2 (2)*	
PLPV (total) = 23/105 (22%)	17/105 (16%)	6/105 (6%)	
Other renal tumors (n = 16)	*SDHB (2), NF1 (1)*	*BARD1 (1)*	
PLPV (total) = 4/16 (25%)	3/16 (19%)	1/16 (6%)	
Thyroid carcinoma/adenoma (n = 85)	*DICER1 (7), RET (3), APC (3), PTEN (2), RB1 (1)*	*PALB2 (1), MUTYH (3), CHEK2 (1), RAD51C (1)*	
PLPV (total) = 22/85 (26%)	16/85 (19%)	6/85 (7%)	
Neuroblastoma/ ganglioneuroma (n = 50)	*PHOX2B (1), PTPN11 (1)*	*CHEK2 (3), RAD51C (1)*	
PLPV (total) = 6/50 (12%)	2/50 (4%)	4/50 (8%)	

Gonadal tumors (n = 29)	*DICER1 (3), PTEN (1), SUFU (1)*	*CHEK2 (1), MUTYH (1)*	
PLPV (total) = 7/29 (24%)	5/29 (17%)	2/29 (7%)	
Neuroendocrine tumors (n =15)	*VHL (3), SDHB (2), NF1 (1)*	0	
PLPV (total) = 7/15 (47%)	7/15 (47%)	0	
Extra-gonadal and extra-cranial germ cell tumors (n = 8)	*PTEN (1)*	*BRCA2 (1), CHEK2 (1), MUTYH (1)*	
PLPV (total) = 4/8 (35%)	1/8 (12%)	3/8 (23%)	
Retinoblastoma (n = 5)	*RB1 (4)*	0	
PLPV (total) = 4/5 (80%)	4/5 (80%)	0	
Breast tumors (n= 30)	*PTEN (3)*	*BRCA1 (1), BRCA2 (1), ATM (1), CHEK2 (1)*	
PLPV (total) = 7/30 (23%)	3/30 (10%)	4/30 (13%)	
Hepatic tumors (n = 5)	*TP53 (1)*	*ATM (1)*	
PLPV (total) = 2/5 (40%)	1/5 (20%)	1/5 (20%)	
Fibrodysplasia (n = 3)	0	0	*ACVR1 (3)*
PLPV (total) 3/3 (100%)	0	0	3/3 (100%)
Total patients (n = 886)	24 genes	16 genes	2 genes
PLPV (total) 186/886 (20.5%)	126 /886 (14.2%)	56/886 (6.3%)	4/886 (0.5%)
