# Supplementary material for: Clinical Impact of Germline Multigene Sequencing in Pediatric Cohorts with a Wide Spectrum of Neoplasms
Source: Int J Mol Sci. 2026 Jul 18;27(14):6395. doi: 10.3390/ijms27146395 (PMC13410190; doi:10.3390/ijms27146395)
Supplement: Supplementary file 1 [file ijms-27-06395-s001.zip › ijms-4377847-supplementary/Table S9. Regression Analysis Report.pdf]

📊 Regression Analysis Results

Date: 2026-06-30 19:45:02

Total patients: 886

With mutations: 126 (14.2%)

Without mutations: 760

📋 Descriptive Statistics by Tumor Group

Tumor Group	N total	N mut	% mut	N Jong+	% Jong+	N mut (Jong+)	% mut (Jong+)	N mut (Jong−)	% mut (Jong−)
CNS	179	30	16.8%	81	45.3%	30	37.0%	0	0.0%
Bone tumors	119	13	10.9%	16	13.4%	9	56.2%	4	3.9%
Embryonal tumors	155	21	13.5%	57	36.8%	18	31.6%	3	3.1%
Endocrine tumors	94	20	21.3%	52	55.3%	18	34.6%	2	4.8%
Hematological malignancies	151	2	1.3%	9	6.0%	2	22.2%	0	0.0%
Others	108	19	17.6%	66	61.1%	18	27.3%	1	2.4%
Soft-tissue tumors	80	21	26.2%	31	38.8%	18	58.1%	3	6.1%

📊 Multicollinearity Check (VIF)

Max VIF: Model 1 = 3.523, Model 2 = 3.503

Result: ✅ No multicollinearity detected (all VIF < 5)

VIF — Model 1 (Jongmans)

Variable	VIF	Interpretation
Cohort	3.522570	✅ OK (< 5)

Variable	VIF	Interpretation
Age	3.433776	✅ OK (< 5)
Classification_Others	1.805873	✅ OK (< 5)
Classification_Endocrine tumors	1.804737	✅ OK (< 5)
Jongmans	1.759473	✅ OK (< 5)
Sex	1.731946	✅ OK (< 5)
Classification_Bone tumors	1.577334	✅ OK (< 5)
Classification_Embryonal tumors	1.434119	✅ OK (< 5)
Classification_Hematological malignancies	1.357275	✅ OK (< 5)
Classification_Soft-tissue tumors	1.349851	✅ OK (< 5)

VIF — Model 2 (Total score)

Variable	VIF	Interpretation
Cohort	3.502626	✅ OK (< 5)
Age	3.430840	✅ OK (< 5)
Classification_Endocrine tumors	1.796445	✅ OK (< 5)
Classification_Others	1.794885	✅ OK (< 5)
Sex	1.734986	✅ OK (< 5)
Total_score	1.589802	✅ OK (< 5)
Classification_Bone tumors	1.577079	✅ OK (< 5)
Classification_Embryonal tumors	1.430262	✅ OK (< 5)
Classification_Hematological malignancies	1.357568	✅ OK (< 5)
Classification_Soft-tissue tumors	1.349713	✅ OK (< 5)

📊 AIC Table

Model	Parameters	Log-Likelihood	AIC	BIC
Model 1 (Jongmans)	11	-255.755	533.510	586.164
Model 2 (Total score)	11	-228.670	479.339	531.993

📊 Performance Metrics (at optimal threshold)

** Sensitivity, Specificity, PPV, NPV, F1 calculated at Youden's optimal threshold.*

Metric	Model 1	Model 2
AUC	0.8600	0.8971
Sensitivity	0.8968	0.8889
Specificity	0.7421	0.7592
Accuracy	0.7641	0.7777
PPV	0.3657	0.3797
NPV	0.9775	0.9763
F1	0.5195	0.5321

📊 AUC with 95% Confidence Intervals (apparent AUC)

** AUC calculated on the entire dataset (apparent AUC). 95% CI estimated via bootstrap (2000 resamples). p-value tests whether AUC > 0.5.*

Model	Apparent AUC	95% CI Lower	95% CI Upper	Std	p-value
Model 1 (Jongmans)	0.8600	0.8290	0.8878	0.0152	<0.001
Model 2 (Total score)	0.8971	0.8674	0.9226	0.0138	<0.001

📊 10-Fold Cross-Validation Results

** Mean AUC from 10-fold stratified cross-validation with standard deviation, minimum and maximum values.*

Model	Mean AUC (10-fold CV)	SD	Min AUC	Max AUC
Model 1 (Jongmans)	0.8491	0.0427	0.7489	0.9090
Model 2 (Total score)	0.8860	0.0453	0.7966	0.9555

📋 Model 1: Jongmans (binary predictor)

> **AIC:** 533.510 | **Apparent AUC:** 0.8600

Variable	OR	95% CI Lower	95% CI Upper	p-value	Significant
Jongmans (Yes vs No)	24.310	12.544	47.112	<0.001	✅ Yes
Sex (Male vs Female)	0.913	0.577	1.442	0.695	❌ No
Age (per 1 year)	0.986	0.939	1.035	0.568	❌ No
Cohort (PC vs RC)	1.424	0.788	2.573	0.241	❌ No
Bone tumors (vs CNS)	2.533	1.034	6.205	0.042	✅ Yes
Embryonal tumors (vs CNS)	0.879	0.434	1.781	0.720	❌ No
Endocrine tumors (vs CNS)	1.048	0.483	2.273	0.905	❌ No
Hematological malignancies (vs CNS)	0.308	0.066	1.435	0.134	❌ No
Others (vs CNS)	0.678	0.325	1.413	0.300	❌ No
Soft-tissue tumors (vs CNS)	2.553	1.169	5.576	0.019	✅ Yes

📋 Model 2: Total score (continuous predictor)

AIC: 479.339 | **Apparent AUC:** 0.8971

Variable	OR	95% CI Lower	95% CI Upper	p-value	Significant
Total score (per 1 point)	10.694	7.095	16.120	<0.001	✅ Yes
Sex (Male vs Female)	0.878	0.530	1.456	0.614	❌ No
Age (per 1 year)	1.001	0.949	1.056	0.970	❌ No
Cohort (PC vs RC)	1.389	0.729	2.649	0.318	❌ No
Bone tumors (vs CNS)	4.048	1.570	10.437	0.004	✅ Yes
Embryonal tumors (vs CNS)	1.087	0.479	2.463	0.842	❌ No
Endocrine tumors (vs CNS)	1.822	0.759	4.377	0.179	❌ No
Hematological malignancies (vs CNS)	0.366	0.071	1.891	0.230	❌ No
Others (vs CNS)	0.891	0.373	2.128	0.794	❌ No
Soft-tissue tumors (vs CNS)	4.825	2.059	11.308	<0.001	✅ Yes

📊 Key Results

DeLong test: diff = +0.0370, z = 0.0000, p = 1.000000

✅ **Difference is statistically significant** (p < 0.05)

📊 ROC Curves

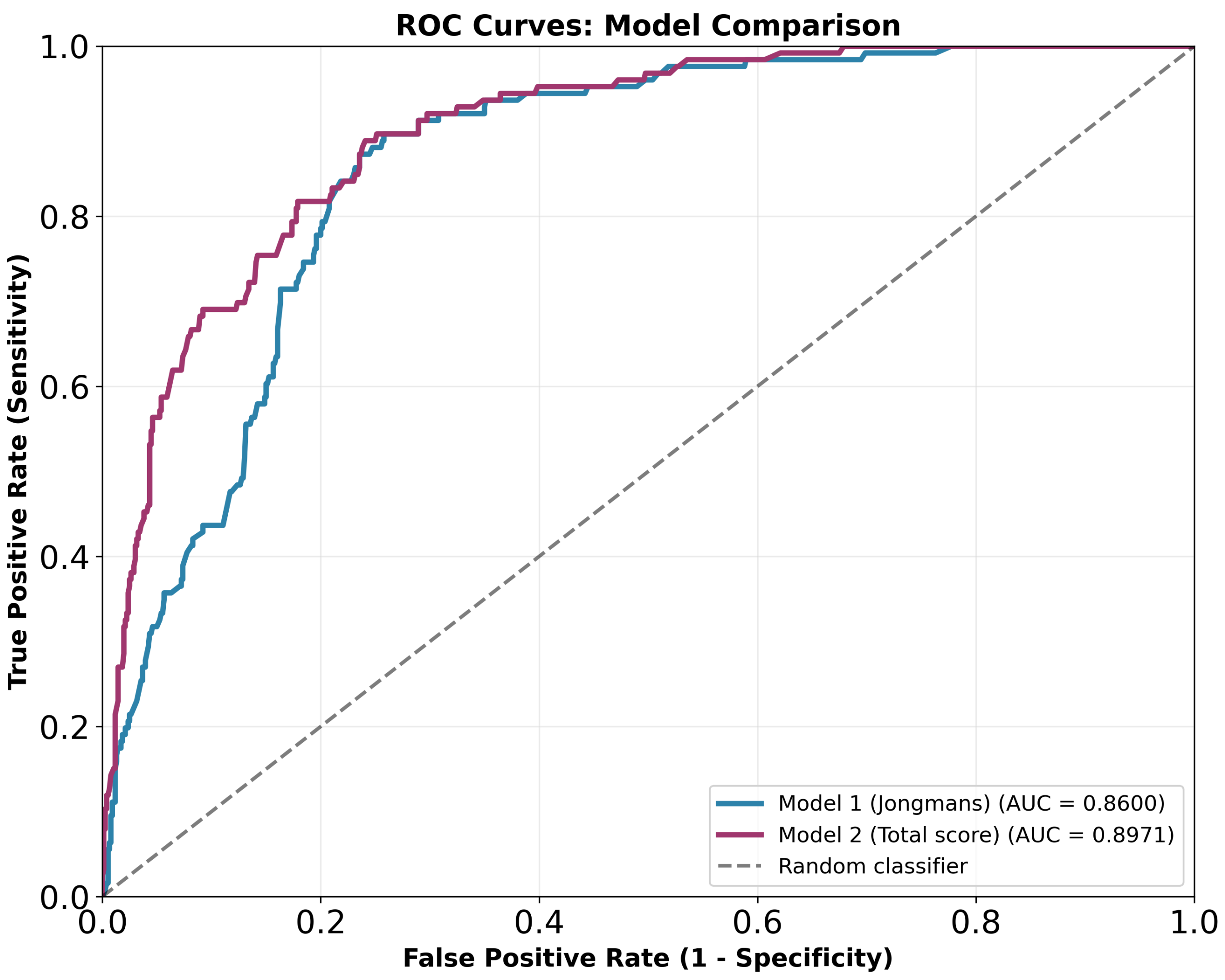

📊 Forest Plot — Model 1 (Jongmans)

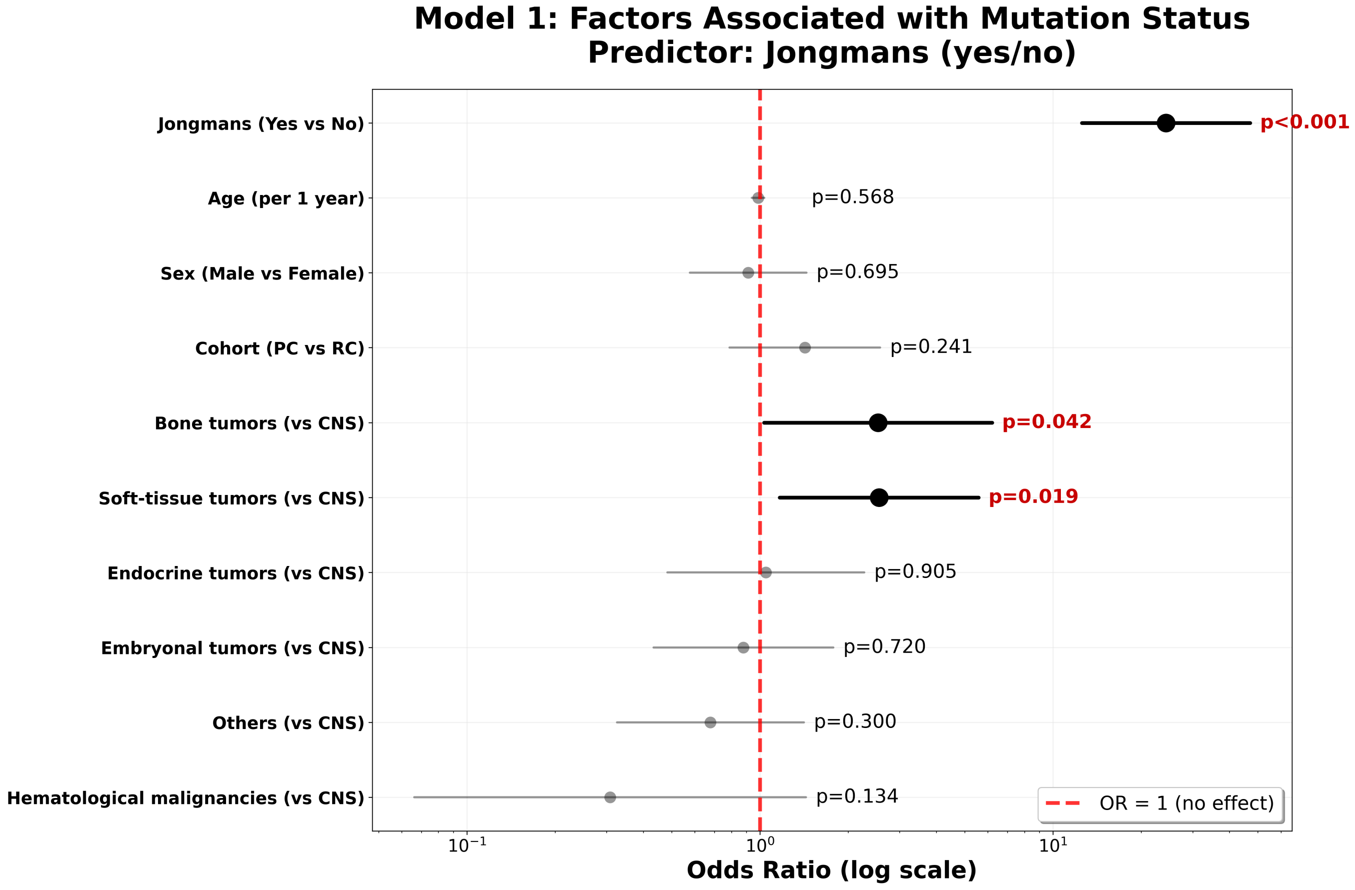

📊 Forest Plot — Model 2 (Total score)

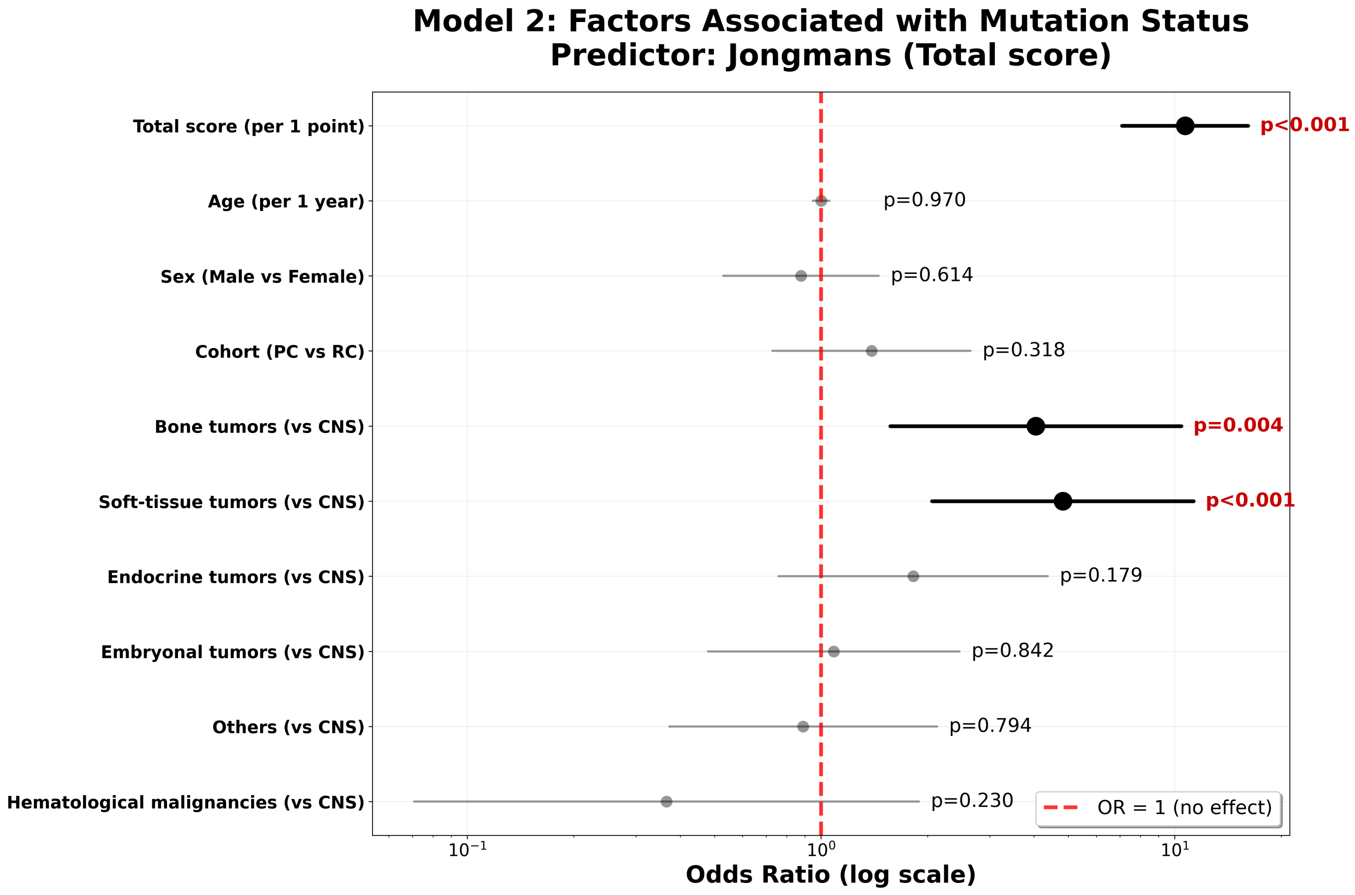

Report generated: 2026-06-30 19:45:02

Python · statsmodels · scikit-learn · matplotlib
