# Supplementary material for: Clinical Impact of Germline Multigene Sequencing in Pediatric Cohorts with a Wide Spectrum of Neoplasms
Source: Int J Mol Sci. 2026 Jul 18;27(14):6395. doi: 10.3390/ijms27146395 (PMC13410190; doi:10.3390/ijms27146395)
Supplement: Supplementary file 1 [file ijms-27-06395-s001.zip › ijms-4377847-supplementary/Tables S6, S7, S8.pdf]

Table S6. Comparison of mutation rate in CPS genes between prospective and retrospective cohorts (Fisher's exact test, *p** < 0.05 is significant).

**Neoplasm type**	**Cohort**		**OR; 95% CI; *p*-value**
	**Prospective**	**Retrospective**	
Total sample	130/477 (27%)	52/409 (13%)	**2.6; 1.8 – 3.7; < 0.0001***
CNS tumors	22/89 (25%)	19/90 (21%)	1.2; 0.6 – 2.5; 0.6
Neuroblastomas	1/28 (4%)	3/22 (8%)	0.2; 0.02 – 2.4; 0.3
Rhabdomyosarcomas	12/44 (27%)	4/16 (25%)	1.1; 03 – 4.2; 1.0
Nephroblastomas	19/84 (23%)	4/36 (11%)	2.3; 0.7 – 7.5; 0.2

Table S7. Frequencies of PLP variants identified by clinical exome or targeted panel (415 genes) sequencing (Fisher's exact test, p < 0.05 is significant).

**Method**	**All patients**	**Patients with PLPV**	**%**
**All variants**			
CES	390	74	18.9%
Panel	496	102	20.5%
18.9% vs. 20.5%; OR = 0.9; 95% CI: 0.6 – 1.3; *p* = 0.6			
**Solid tumors**			
CES	249	67	26.9%
Panel	487	101	20.7%
26.9% vs. 20.7%; OR = 1.4; 95% CI: 0.9 – 2.0; *p* = 0.06			
**Hematologic neoplasms**			
CES	140	7	5%
Panel	10	1	10%
5% vs. 10%; OR = 0.5; 95% CI: 0.05 – 4.3; *p* = 0.43			
**Causative variants**			
CES	390	51	13.1%
Panel	496	78	15.7%
13.1% vs. 15.7%; OR = 0.8; 95% CI: 0.5 – 1.2; *p* = 0.3			
**Solid tumors**			
CES	249	50	20.0%
Panel	487	77	15.8%
20.0% vs. 15.8%; OR = 1.3; 95% CI: 0.9 – 2.0; *p* = 0.1			
**Hematologic neoplasms**			
CES	140	1	0.7%
Panel	10	1	10%
0.7% vs. 10.5%; OR = 0.06; 95% CI: 0.01 – 1.1; *p* = 0.12			

Table S8. Contribution of clinical exome sequencing (CES) and targeted 415-gene panel into total diagnostic yield

Parameter	CES (n=390)	Panel (415 genes) (n=496)
**PLP variants in cancer predisposition genes (n=176)**	74/176 (42%)	102/176 (57.9%)
**Gene spectrum**	*ACVR1, APC, ATM, BLM, BRCA1, BRCA2, CHEK2, DICER1, EXT1, MSH2, MSH3, MUTYH, NBN, NF1, NF2, PHOX2B, PKHD1, PMS2, PTEN, RAD51C, RB1, REST, SDHB, SMARCAL1, SMARCB1, SUFU, TP53, TRIM28, WT1* (n=29)	*ACVR1, APC, ATM, BARD1, BRCA1, BRCA2, CHEK2, DDX41, DICER1, MSH2, MSH6, MUTYH, NBN, NF1, PALB2, POLE, PRKAR1A, PTCH1, PTEN, PTPN11, RAD51C, RB1, RECQL4, REST, RET, SDHB, SMARCA4, SMARCB1, TP53, TRIM28, VHL, WT1* (n=32)
**Mean coverage**	150×	250×
**Mean number of variants identified per sample**	10973	2356,4
**Genes with insufficient coverage (<20×)**	0	0
